# Stiffness calibration of qPlus sensors at low temperature through thermal noise measurements

**DOI:** 10.3762/bjnano.15.50

**Published:** 2024-05-23

**Authors:** Laurent Nony, Sylvain Clair, Daniel Uehli, Aitziber Herrero, Jean-Marc Themlin, Andrea Campos, Franck Para, Alessandro Pioda, Christian Loppacher

**Affiliations:** 1 Aix Marseille University, CNRS, IM2NP, UMR 7334, 13397 Marseille, France; 2 SPECS Zürich GmbH, Technoparkstrasse 1, 8005 Zürich, Switzerland; 3 Aix Marseille University, CNRS, Centrale Marseille, FSCM (FR1739), CP2M, 13397 Marseille, France

**Keywords:** low temperature, non-contact atomic force microscopy, qPlus sensors, quartz tuning fork, stiffness calibration, thermal noise, ultrahigh vacuum

## Abstract

Non-contact atomic force microscopy (nc-AFM) offers a unique experimental framework for topographical imaging of surfaces with atomic and/or sub-molecular resolution. The technique also permits to perform frequency shift spectroscopy to quantitatively evaluate the tip–sample interaction forces and potentials above individual atoms or molecules. The stiffness of the probe, *k*, is then required to perform the frequency shift-to-force conversion. However, this quantity is generally known with little precision. An accurate stiffness calibration is therefore mandatory if accurate force measurements are targeted. In nc-AFM, the probe may either be a silicon cantilever, a quartz tuning fork (QTF), or a length extensional resonator (LER). When used in ultrahigh vacuum (UHV) and at low temperature, the technique mostly employs QTFs, based on the so-called qPlus design, which actually covers different types of sensors in terms of size and design of the electrodes. They all have in common a QTF featuring a metallic tip glued at the free end of one of its prongs. In this study, we report the stiffness calibration of a particular type of qPlus sensor in UHV and at 9.8 K by means of thermal noise measurements. The stiffness calibration of such high-*k* sensors, featuring high quality factors (*Q*) as well, requires to master both the acquisition parameters and the data post-processing. Our approach relies both on numerical simulations and experimental results. A thorough analysis of the thermal noise power spectral density of the qPlus fluctuations leads to an estimated stiffness of the first flexural eigenmode of ≃2000 N/m, with a maximum uncertainty of 10%, whereas the static stiffness of the sensor without tip is expected to be ≃3300 N/m. The former value must not be considered as being representative of a generic value for any qPlus, as our study stresses the influence of the tip on the estimated stiffness and points towards the need for the individual calibration of these probes. Although the framework focuses on a particular kind of sensor, it may be adapted to any high-*k*, high-*Q* nc-AFM probe used under similar conditions, such as silicon cantilevers and LERs.

## Introduction

Since the 2000s, non-contact atomic force microscopy (nc-AFM) has established itself as a scanning probe method for the topographical, chemical, and electrical mapping of the surface of a sample down to the atomic scale [1–3]. When used in an ultrahigh-vacuum (UHV) system and at, or close to, liquid helium temperature (4–10 K, LT UHV), the method allows for the direct characterization of individual molecules with intramolecular contrast [4], opening up the field of studying on-surface reactions [5] or tip-induced chemistry [6].

The method also makes it possible to quantify the interatomic interaction forces that develop between the tip and the surface acquired in spectroscopic data cube modes [7–8] with both high sensitivity and high spatial resolution. Recently, the force sensitivity has been pushed forward, and forces as low as 100 fN have been reported on artificial atoms formed by quantum corrals [9].

In nc-AFM, the probe, whose mechanical behavior may advantageously be compared to that of a one-dimensional simple harmonic oscillator (SHO) of resonance frequency *f*_1_ (flexural fundamental eigenmode) and stiffness *k*_1_, is sinusoidally excited at *f*_1_ by a phase-locked loop (PLL) that also guarantees a constant oscillation amplitude, *A*_1_ [10]. If the tip is far enough from the surface, that is, at distances where the strength of the tip–surface interatomic forces is negligible with respect to the restoring force induced by the excitation, its resonance frequency remains unchanged, *f*_1_. When the tip is in the range of attractive interatomic forces *F*_int_(*r*), that is, for tip–surface separations *r* 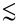 1 nm, non-linear effects modify the oscillator dynamics, which shifts its resonance frequency down to lower values 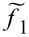 *< f*_1_. The resulting frequency shift Δ*f* = 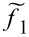 − *f*_1_ *<* 0 is tracked by the PLL and used as the input of the *Z*-controller to form a “topographic image”, which is actually a “constant-Δ*f*” image. Alternatively, the image can also be acquired at constant height, which then forms a local Δ*f* map of the surface. Δ*f* is expressed according to [11–12]:


[1] $$\Delta f\left(z\right)=\frac{f_{1}}{2\pi k_{1}A_{1}}\int_{0}^{2\pi }F_{\mathrm{int}}\left(r_{u}\left(z\right)\right)\cos\left(u\right)\text{d}u,$$

where *r_u_*(*z*) = *z* + *A*_1_(1 − cos(*u*)) is the instantaneous tip–surface position, and *z* is the shortest distance between the tip and the surface during one oscillation cycle. Thus, if *A*_1_ and *k*_1_ are properly calibrated, the interaction force may be quantified, however, through non-trivial inversion procedures [13–16]. The amplitude calibration in nc-AFM using the so-called constant-γ method is well documented and reasonably accurate [16–18], even if recently reported methods seem more accurate [16]. Conversely, it seems that the direct stiffness calibration of nc-AFM probes in UHV and at low temperature has never been reported. Furthermore, because the force sensitivity in nc-AFM critically depends on the mechanical stability of both probe and tip, it seems crucial to perform the probe stiffness calibration in situ, that is, within the LT UHV system, by means of a non-destructive method.

In UHV and at room temperature, nc-AFM experiments are mostly carried out with silicon cantilevers, similar to those used during AFM experiments in air or in liquid. Their stiffness rarely exceeds 100 N/m. In UHV and at low temperature, the use of cantilevers is more tedious because of the required in situ optical detection setup. Nc-AFM experiments are then mostly performed with quartz sensors, essentially implemented according to two geometries: quartz tuning fork (QTF) [19] or length-extensional resonator (LER) [20–22]. The commercial versions of these probes are the qPlus sensor (Scienta-Omicron) [19,23] and the KolibriSensor (SPECS) [24–26], respectively. It is known that these sensors offer several advantages: (i) Their large stiffness (≃1800 N/m for qPlus and ≃540 × 10^3^ N/m for KolibriSensor), much greater than that of silicon cantilevers. It prevents the snap of the tip into contact and enables the use of small oscillation amplitudes (*A*_1_ ≃ 50 pm), which render the probe highly sensitive to the short-range regime of interatomic forces. (ii) Their high quality factor (*Q*, ≃10^5^ in a LT UHV system), which renders the PLL highly sensitive to the frequency tracking. (iii) Their piezoelectric nature, which facilitates the readout of the tip deflection, based on the piezoelectric charge induced by the quartz upon oscillation through a simple *I*/*V*, or charge, preamplifier [27–28], as compared to the heavy optical detection setup required for silicon cantilevers.

Nowadays, the qPlus sensor is the probe that is most commonly used with LT UHV microscopes. This is why we focus on this type of probe in this work. In this design, only one prong of the QTF is fixed [19]. At the extremity of the free prong, a thin, etched wire (usually W or PtIr), less than a millimeter long, is glued, which forms the tip. The tip is electrically connected to an electrode that collects the tunneling current if scanning tunneling experiments are to be performed along with nc-AFM experiments. The qPlus sensors feature a resonance frequency of *f*_1_ ≃ 25 kHz and a most commonly reported stiffness of 1800 N/m [19]. This estimate was first proposed in 2000 [29], following previous works [30–31], and was based on geometric criteria of the sensor that did not consider the influence of the added tip. Thus, this value of the stiffness reported for the early versions of qPlus, which is still used in most of the recent works to perform the frequency shift-to-force conversion (see, e.g., the supplementary material of [8] and [9]), is not necessarily compatible with that of modern ones. Furthermore, because the detailed geometry of each tip is never the same (regarding, e.g., diameter and length), and because it cannot be glued on the prong with high reproducibility (regarding, e.g, mass of glue and location on the prong), the mechanical properties of each sensor must differ in detail. Therefore, the actual stiffness of each probe must differ and has no reason to match a particular predefined value.

The stiffness calibration of silicon cantilevers at room temperature in air, liquid, or UHV by means of destructive and non-destructive methods has been discussed quite extensively in the literature [18,32–60], leading to a set of a dozen distinct approaches. A “global calibration initiative” has even been launched by Sader [58,61]. Conversely, much less references are available for qPlus sensors [62–67], and, among these, none of them deals with the direct stiffness calibration of the probe in a LT UHV system.

The goal of the present work is to propose a framework based on thermal noise measurement to calibrate the stiffness of qPlus sensors operated in a LT UHV system. The concept was introduced by Hutter and Bechhoefer, and Butt et al. in 1993 [33–34], and was further improved by Butt and Jaschke in 1995 [35]. It is based on the measurement of the spectrum of the fluctuations of the free end of the probe excited by thermal noise. The peak of the thermal noise spectrum at the resonance frequency of the probe may be related to its stiffness if the mechanical behavior of the probe can be modeled as that of an equivalent SHO. Our framework combines experimental measurements performed with qPlus sensors in UHV at 9.8 K and numerical simulations of the thermal fluctuations of a SHO under equivalent conditions. The numerical results permit to refine the experimental strategy, which allows us to achieve an uncertainty of 10% maximum in the calibration.

This work is inspired by, and based on, results from the literature, but extends the scope of AFM probes stiffness calibration through thermal noise measurement to very stiff (*k >* 1000 N/m) and large-*Q* (*Q >* 10^5^) probes, such as qPlus sensors. To this end, many theoretical and practical elements are detailed, which usually are either not clearly stated or little discussed in the literature because they are not salient with softer probes, but they become crucial with very rigid and high-*Q* probes in LT UHV.

Finally, we want to stress that although this work treats the particular case of qPlus sensors, the framework can be adapted to any other kind of nc-AFM probes used in LT UHV, including other types of QTFs, silicon cantilevers, and LERs, with the KolibriSensor among the latter.

The paper is organized as follows. The section “Stiffness calibration methods: a brief review” briefly introduces the bibliographic context of the stiffness calibration methods, restricted to the main of our requirements. Section “Framework to the stiffness calibration” details the concepts of stiffness calibration based on thermal noise measurement, along with our assumptions. Section “Numerical simulations” details the numerical approach to the stiffness calibration. Section “Experimental results” presents the experimental results, which are discussed in section “Discussion” before the Conclusion.

Four Supporting Information files support our framework. They contain important results from the literature and are organized to help the reader to follow our developments. Supporting Information File 1 reminds the most salient results of the Euler–Bernoulli model and how it sustains the point-mass SHO equivalence. Supporting Information File 2 reminds fundamental elements of signal processing applied to discrete time signals, which include the power spectral density (PSD), a key tool for the stiffness calibration. Supporting Information File 3 reminds the expression of the thermal noise PSD of a SHO in thermal equilibrium within a thermostat. The PSD of the stochastic thermal force giving rise to the fluctuations of the SHO is derived as well, which is used in the numerical simulations. Supporting Information File 4 discusses the relevance of a digital antialiasing filter on the measured thermal noise PSD.

## Stiffness Calibration Methods: A Brief Review

This section reminds some salient results about stiffness calibration methods reported in the literature, which forms the context of the present study.

**Figure 1:**
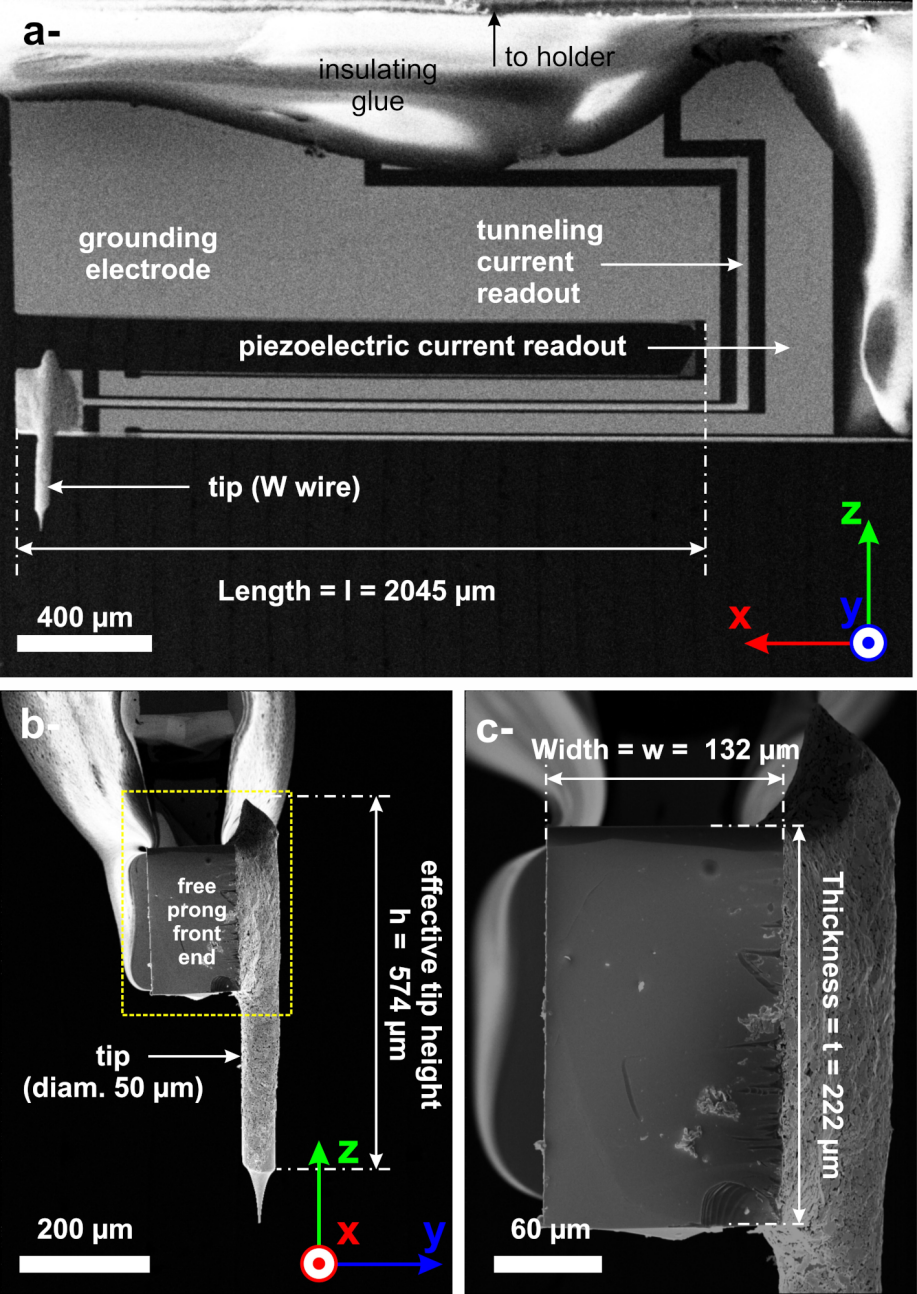
SEM images of the type of qPlus sensor used in this work. All dimensions are estimated with a relative uncertainty of 5%. a- Side view showing the complete geometry of the qPlus sensor. The circuitry of the electrodes is well identifiable. b- Front view of the qPlus free prong showing the tip glued to the right-hand side. c- Magnification of the dotted rectangle shown in b-.

In the following, unless specified otherwise, the word “probe” either means a silicon cantilever or the free prong of a qPlus sensor. The discussion is restricted to probes with a rectangular cross section (length *l*, thickness *t*, width *w* are such that *l* ≫ *t* and *l* ≫ *w*) treated in the Euler–Bernoulli model of the embedded beam, extensively detailed, for example, in [35] (cf. also Supporting Information File 1). The displacement of the probe is assumed to occur vertically (along the *z* axis, as defined in Figure 1) and to be small with respect to all its dimensions (elastic deformation only). The word “deflection” means the displacement that takes place at the free end of the probe with respect to its equilibrium position *z* = 0. Torsional effects are not accounted for, which is justified in the section “Framework to the stiffness calibration” (cf. subsection “Experimental context”).

Since we focus on the stiffness calibration in UHV, we also restrict the context to cases where the hydrodynamic function of the fluid surrounding the probe [38], if described in the model, plays no role.

Following Burnham’s classification [40], we essentially focus on two categories of non-destructive calibration methods, referred to as “geometric” and “thermal” methods.

### Geometric methods

Geometric methods permit to calculate the stiffness of the probe from its dimensions and the mechanical properties of its constitutive material. When the load is applied at the free end of the probe, its static stiffness is given by:


[2] $$k_{\text{s}}=\frac{Ewt^{3}}{4l^{3}},$$

where *E*, *w*, *t*, and *l* are Young’s modulus, width, thickness, and length of the probe, respectively. Cleveland et al. early exploited this concept to determine the stiffness of soft levers [32]. But these authors, as well as Sader et al. [38,68], also proposed a calibration method of the static stiffness based on the measurement of the unloaded resonant frequency of the probe flexural fundamental eigenmode (*f*_1_), whose mass *m*_probe_ is to be estimated then. In this framework, the probe is assumed to behave as an equivalent SHO; then, the static stiffness is derived from *f*_1_ according to: 
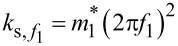
. The quantity 
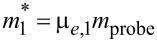
 is the effective mass of the fundamental eigenmode of the probe; *m*_probe_ = ρ*wtl* is calculated from the density ρ, thickness *t*, and plan view dimensions (length *l* and width *w*) of the probe. The quantity μ*_e,_*_1_ is the probe’s normalized effective mass of the fundamental eigenmode, taking the value μ*_e,_*_1_ ≃ 0.2427 for *l*/*w >* 5 [32,38] (cf. also Supporting Information File 1). Because the experimental determination of the length of the probe is prone to less error than that of its thickness (*l* ≫ *t*), Cleveland et al. and Sader et al. removed the thickness dependence from Equation 2 and came to an equivalent expression for 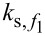:


[3] $$k_{\text{s},f_{1}}=2w{\left(\pi lf_{1}\right)}^{3}\sqrt{\frac{\rho^{3}}{E}}.$$

As discussed by Burnham et al. [40], for rectangular probes with a stiffness of ≃1 N/m, Cleveland/Sader’s calibration methods agree within 17% of the manufacturer’s nominal value.

In 2012, Lübbe et al. extended Cleveland/Sader’s approach to the Euler–Bernoulli model and derived an expression giving the static stiffness of the probe from the resonance frequency of any of its flexural eigenmodes [51]:


[4] $$k_{\text{s},f_{n}}=\frac{2w{\left(\pi lf_{n}\right)}^{3}}{\alpha_{n}^{6}}\sqrt{\frac{12^{3}\rho^{3}}{E}},$$

where α*_n_* is a term occurring in functions that define the Euler–Bernoulli model used to describe the probe oscillation. α*_n_* is the solution of a so-called dispersion relation, written as [35,48–49,55]:


[5] $$1+\cos\left(\alpha_{n}\right)\cosh\left(\alpha_{n}\right)=0,$$

leading to α_1_ = 1.875, α_2_ = 4.694, α_3_ = 7.864, …, α*_n_* ≃ (*n* − 1/2)π.

The latter formalisms consider a homogeneous probe without influence of the added mass due to the presence of the tip at its free end (unloaded case). This added mass changes the eigenmodes geometry, though. This results in a change of the value of the constant α*_n_* of each eigenmode. Lozano et al. [49], Lübbe et al. [51], and Yamada et al. [69] have addressed the issue of the tip mass correction in the Euler–Bernoulli model. To this end, an extended probe oscillation model is used [70], which leads to a new equation for α*_n_* (loaded case), now written 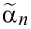 in order to not confuse it with the solution of the unloaded case:


[6]

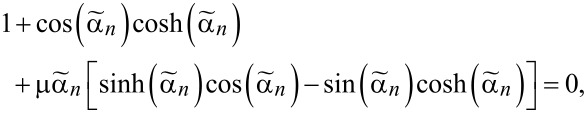



where μ = *m*_tip_/*m*_probe_ is the ratio between the tip mass and the probe mass. Hence, μ must now be established before obtaining the value of 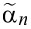.

In their work, Lübbe et al. [51] point out that the direct stiffness calibration from the probe dimensions yields values with an uncertainty of ±25% as the result critically depends on the probe thickness, which is difficult to determine experimentally. But the uncertainty is reduced to ±7% when the measured fundamental eigenfrequency is included in the calculation and a tip mass correction is applied.

### Thermal noise methods

Thermal methods are based on the measurement of the probe’s thermal fluctuations when it is in thermal equilibrium within a thermal bath [35,40,43–44,46–48,55–57,59–60,71]. The influence of thermal noise on a system has first been investigated by Nyquist and Johnson in 1928 with electric resistors [72–73]. Their seminal work has later been formalized by the linear response theory and the fluctuation–dissipation theorem (FDT) [74–75], establishing a connection between fluctuations about equilibrium and the response of a system to external forces upon its susceptibility (or response function).

Thermal energy and probe fluctuations are linked by the equipartition theorem, which states that the energy transferred from a thermal bath to a dynamic system equals *k*_B_*T*/2 for each of its degrees of freedom, *k*_B_ being the Boltzmann constant and *T* the temperature of the thermostat. Here, as discussed in [35,48,56], the probe is described as an equivalent point-mass SHO that features stochastic deflections of its free end along the vertical *z* axis over time due to thermal noise, forming a signal *z*_th_(*t*). The equipartition theorem is written as:


[7] $$\frac{1}{2}k_{\text{B}}T=\frac{1}{2}k_{\text{s}}\langle z_{\text{th}}^{2}\rangle ,$$

where ⟨⟩ represents a virtually infinite time averaging. The quantity


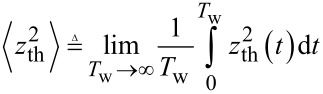


is the power of the probe fluctuations (mean quadratic deflections) induced by thermal noise over time. For a qPlus sensor of stiffness *k*_s_ ≃ 1800 N/m at *T* = 9.8 K, the rms deflection induced by thermal noise is 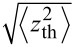 ≃ 270 fm.

As discussed early by Butt and Jaschke [35] and explicitly measured by others [48,55], the rigorous analysis of the thermal fluctuations is to be performed in terms of modal decomposition of the probe deflections over its eigenmodes. Then, the total deflection of the probe’s free end due to thermal fluctuations results from the superposition of the deflections of equivalent SHOs embodying the eigenmodes of the probe, which are assumed to be independent [49]. Because of their high quality factors, the former assumption is particularly valid for qPlus sensors (cf. also Supporting Information File 1). Thus:


[8] $$\langle z_{\text{th}}^{2}\rangle =\sum_{n=1}^{+\infty }\langle z_{\text{th},n}^{2}\rangle ,$$

where 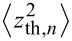 is the power of the thermal fluctuations of the *n*-th flexural eigenmode (resonance frequency *f_n_*, quality factor *Q_n_*, and stiffness *k_n_*), and the summation represents all eigenmodes of the probe. Under the assumption of thermal equilibrium, the thermal noise-induced deflection of each eigenmode follows the equipartition theorem, such that:


[9] $$\frac{1}{2}k_{\text{B}}T=\frac{1}{2}k_{n}\langle z_{\text{th},n}^{2}\rangle .$$

Upon proper normalization of the solution functions of the Euler–Bernoulli model, the modal stiffness *k_n_* of an unloaded probe may be connected to the static one *k*_s_ (or equivalently 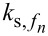) according to [35,76]:


[10] $$\frac{k_{n}}{k_{\text{s}}}=\frac{\alpha_{n}^{4}}{12}.$$

Thus, for the fundamental eigenmode of an unloaded probe (α_1_ = 1.875), 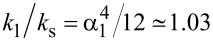.

Combining Equation 8, Equation 9, and Equation 10 yields:


[11] $$\langle z_{\text{th}}^{2}\rangle =k_{\text{B}}T\sum_{n=1}^{+\infty }\frac{1}{k_{n}}=\frac{k_{\text{B}}T}{k_{\text{s}}}\sum_{n=1}^{+\infty }\frac{12}{\alpha_{n}^{4}}.$$

Equation 11 is similar to Equation 7 if the summation is performed over all the eigenmodes of the probe (
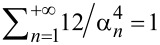
, cf. [35,77]).

From an experimental point of view, the number of accessible eigenmodes (*m*) is limited because the detection bandwidth of the fluctuations is restricted. Then, the relative error introduced in the estimated static stiffness is 
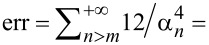
 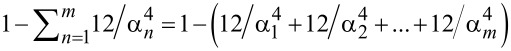, which may be estimated. For instance, restricting the detection bandwidth to the fundamental eigenmode of an unloaded probe (α_1_ = 1.875) sets *m* = 1 and 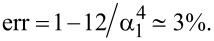 In other words, the modal stiffness *k*_1_ of the fundamental eigenmode of an unloaded probe exceeds the static stiffness *k*_s_ by 3%, or equivalently, 97% of the thermal fluctuations are due to the probe’s fundamental eigenmode.

Equation 9 states that the measurement of the thermal fluctuations of the deflection of the *n*-th eigenmode over an arbitrary long time interval might allow us to derive the corresponding modal stiffness. But this is not feasible in practice because of measurement noise, which usually exceeds thermal noise. There are several origins to measurement noise. For qPlus sensors, two main noise sources may be considered: the preamplifier, which converts the piezoelectric current of the QTF into a scalable voltage signal, and the subsequent analog/digital converter (ADC), which converts the analog signal into a digital signal to be processed by the digital control unit of the microscope. It is difficult to quantify the strength of those sources with respect to that of the thermal noise based on the time trace of the fluctuations as it gives no idea on how the noise is spectrally spread within the system. This is why the analysis of noisy signals is rather performed from their PSD. The PSD *S_z_*(*f*) of an analog signal *z*(*t*) featuring thermal fluctuations with measurement noise is defined according to:


[12] $$\langle z^{2}\rangle =\int_{-\infty }^{\infty }S_{z}\left(f\right)\text{d}f.$$

It is also usually assumed that thermal noise and measurement noise are uncorrelated. Thus, noting the power of the measurement noise:


[13] $$\langle z_{\text{mn}}^{\text{2}}\rangle =\int_{-\infty }^{\infty }S_{z_{\text{mn}}}\left(f\right)\text{d}f,$$

the power of the measured thermal noise probe deflections is such that:


[14] $$\langle z^{2}\rangle =\langle z_{\text{th}}^{\text{2}}\rangle +\langle z_{\text{mn}}^{\text{2}}\rangle .$$

The power of the thermal fluctuations without measurement noise is ultimately given by integration of a quantity we name the thermal noise PSD (tn-PSD) 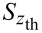
(*f*), according to:


[15] $$\langle z_{\text{th}}^{\text{2}}\rangle =\int_{-\infty }^{\infty }S_{z_{\text{th}}}\left(f\right)\text{d}f=\int_{-\infty }^{\infty }\left[S_{z}\left(f\right)-S_{z_{\text{mn}}}\left(f\right)\right]\text{d}f.$$

Technically, the PSD is defined from the Fourier transform 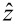
(*f*) of the time trace of the signal *z*(*t*) forming the Fourier pair *z*(*t*) 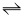 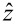
(*f*), according to:


[16] $$S_{z}\left(f\right)=\underset{T_{w}\to \infty }{\lim}\frac{1}{T_{w}}{\left|\hat{z}\left(f\right)\right|}^{2}.$$

Thus, if the measurement noise PSD 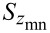
(*f*) is quantified, the quantity 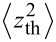 depicting the thermal fluctuations of the probe can be estimated from the measurement of *S_z_*(*f*) (Equation 15) and, thus, also the probe stiffness (Equation 7).

Because the Fourier transform is intrinsically two-sided (*f* ∈ ℝ), the integration in Equation 12, Equation 13, and Equation 15 spreads from −∞ to +∞. The two-sided representation of the DFT forms a strict, self-consistent, mathematical background; however, in the case of the PSD, its one-sided representation is preferred (*f* ∈ [0;+∞[). In addition, the observables that are measured from stochastic signals usually relate to rms values (e.g., V_rms_). It is therefore preferable to express their corresponding PSD from the rms value of their Fourier transform. Supporting Information File 2 explicitly details the connection between the two-sided expression of the PSD and that of the one-sided rms PSD (cf. Supporting Information File 2, Equations S14 and S15). In the following, we will only use the spectral expression/representation of the one-sided rms PSD of the signal *z*_th_(*t*), which is defined for *f* ≥ 0 only. Unlike in Supporting Information File 2, the “rms” superscript will be systematically omitted in the notations in order to lighten them, but it is maintained in the units.

A large part of the thermal fluctuations stems from the probe’s fundamental eigenmode. Thus, it is interesting to compare 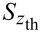
(*f*) to the formal expression of the one-sided rms tn-PSD of an equivalent SHO (resonance frequency *f*_1_, quality factor *Q*_1_, and stiffness *k*_1_), which is established in Supporting Information File 3 (cf. Equation S8). This quantity is written as:


[17] $$S_{\text{SHO}}\left(u=f/f_{1}\right)=\frac{2k_{\text{B}}T}{\pi k_{1}Q_{1}f_{1}}\frac{1}{{\left[1-u^{2}\right]}^{2}+\frac{u^{2}}{Q_{1}^{2}}}.$$

The function exhibits a resonance for *f* = *f*_1_ (*u* = 1), and then:


[18] $$S_{\text{SHO}}\left(u=1\right)=\frac{2k_{\text{B}}TQ_{1}}{\pi k_{1}f_{1}}.$$

Equation 15, Equation 16, Equation 17, and Equation 18 form the analytical framework for the analysis of the thermal fluctuations, which ultimately allows for the probe stiffness calibration from Equation 7. They are used according to three methodological approaches, which all are reported in the literature:

- **Method 1**: 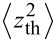 is derived by integration of the tn-PSD 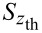
(*f*) (Equation 15), that is, the as-measured thermal noise PSD *S_z_*(*f*) corrected from its measurement noise 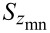
(*f*). *S_z_*(*f*) may either be measured with a properly calibrated spectrum analyzer or derived from the Fourier transform of the time trace of the thermal fluctuations (Equation 16) [48]. Depending on the acquisition bandwidth, *k*_s_ may be estimated from Equation 10 or Equation 11.
- **Method 2**: The stiffness of the probe’s fundamental eigenmode *k*_1_ may as well be fitted from 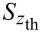
(*f*) from the tn-PSD of the SHO (Equation 17) [56], provided that (i) the probe’s mechanical behavior satisfactorily compares to that of an equivalent SHO, and (ii) resonance frequency *f*_1_ and quality factor *Q*_1_ of the probe are known. Then, *k*_s_ may be derived from Equation 10.
- **Method 3**: *k*_1_ may be directly estimated from the maximum of 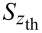
(*f*) (Equation 18) [40], provided that *f*_1_ and *Q*_1_ are known. Then, *k*_s_ may be derived from Equation 10.

These methods all require a good estimate of the measurement noise PSD 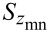
(*f*), otherwise the estimated stiffness will be uncertain (cf. hereafter).

### Other non-destructive methods

Finite element method (FEM) modeling has been applied successfully to calibrate the stiffness of both silicon cantilevers [59,76,78–82] and qPlus sensors [18,65]. For qPlus sensors, however, FEM does not offer a generic approach. Indeed, as presented in the Introduction, the fact whether the sensors are custom-made or commercial, the tip shape (nature, diameter, and length of the wire), the precise location where it is glued on the free prong, along with the nature and quantity of glue used to hold the wire, imply a large range of geometric parameters, which ultimately influence the resulting stiffness of the probe. Falter et al. pointed out this issue [65], and the authors outlined the urge of stiffness calibration for each sensor. The main conclusion of their FEM modeling shows quantitative agreement with the beam formula (Equation 2) if the beam origin is shifted to the position of zero stress onset inside the tuning fork base; however, there was a systematic overestimation of the experimental stiffness due to the tip gluing geometry.

In the 2000s, Rychen et al. proposed an approach to the calibration of the modal stiffness of QTFs used below 4.2 K and at 5 mbar [62–63]. The method is based on the measurement of the admittance of the piezoelectric current produced by the fork upon oscillation. The authors fitted that quantity with a Butterworth–Van Dyke-type electrical equivalence, and they put in relation the fitted electrical parameters with those of an equivalent mechanical SHO. This approach is valuable as it is performed in situ (however here not in UHV) and is non-destructive. However, it requires the precise knowledge of the piezoelectric constant of the quartz, and, with current qPlus designs, it was shown that the Butterworth–Van Dyke equivalent circuit failed at describing all their features [83].

## Framework to the Stiffness Calibration

### Methodology

Our experimental results are interpreted with the help of numerical simulations, but experimental and numerical approaches rely both on the same methodology.

Our framework to the stiffness calibration consists in processing the time trace of the qPlus thermal fluctuations to extract the quantity 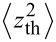. To this end, the time signal of the thermal fluctuations including, or not (in the case of numerical simulations), measurement noise, is acquired over a windowing duration *T*_w_. This process is repeated to form a statistic set of *M* time traces of the fluctuations (*M*_exp_ ≥ 500, *M*_num_ ≥ 60). Then, the properly normalized one-sided rms PSD spectrum of each trace is calculated. The *M* rms PSD spectra are ultimately averaged resulting in a single final spectrum.

The value of the stiffness is then deduced according to the methods 1–3 described before. However, as discussed in detail by Cole [46] or Sader et al. [53], in the case of noisy signals like the tn-PSD, the use of non-linear least-squares fits, such as those required in method 2, is problematic. Their convergence and the accuracy of the fit coefficients may depend on type of noise, type of fit functional, minimization algorithm, and number of coefficients to fit along with their boundary conditions and may lead to erroneous results. Because some of these difficulties were faced when processing our data, we do not use method 2 for the experimental stiffness calibration and restrict the analysis to methods 1 and 3.

### Experimental context

The experimental results presented in this work have been acquired with a closed-cycle UHV SPM Infinity microscope from Scienta-Omicron, operated at 9.8 K. We use commercial qPlus sensors purchased from Scienta-Omicron.

Scanning electron microscopy (SEM) pictures of one of these probes are shown in Figure 1. SEM analysis was performed with a Zeiss GeminiSEM 500 ultrahigh-resolution FESEM at 15 kV. Secondary electron detection was used for imaging. At 15 kV, the resolution is 0.6 nm. Energy-dispersive X-ray spectroscopy (EDS) chemical analyses have been performed too, for which an EDAX Octane Silicon Dri Detector (129 eV energy resolution for manganese) coupled to the SEM was used at 15 kV. A large side view (cf. Figure 1a) shows the overall probe geometry. The qPlus sensors we use feature QTFs whose prong geometry is asymmetric. The sensor is glued by means of an insulating epoxy glue (white areas) on a massive metallic holder, not visible in the figure, that is mechanically clamped into the scanning piezo featuring a ring electrode for mechanical excitation. The QTF surface features a set of three metallic electrodes evaporated on it. Their chemical composition has been characterized by EDS as consisting of a ≃200 nm thick layer of Au on a thinner chromium layer to favor the adhesion and wetting of Au. The massive electrode is for grounding. The two thinner ones, running along the free prong, are for the piezoelectric current and tunneling current readouts. The free prong is *l* = (2045 ± 100) μm long. The tip, indicated at the end of the free prong, consists of a W wire that is 50 μm in diameter, better visible in Figure 1b. It is glued with a conducting epoxy, visible in the pictures, on the side of the free prong, near its end. With this asymmetry, oscillations due to the first torsional eigenmode of the qPlus sensor might occur and perturb the detection of the thermal motion due to the first bending mode. However with regular QTFs, the first torsional eigenmode is expected to be above, or near, our sampling frequency of ≃155 kHz [84] (cf. subsection “Acquisition parameters of the thermal fluctuations as a discrete time signal”). The torsional resonance is, therefore, far away from our considered frequency range, and torsional effects should not influence, to a large extent, our measurements. The W wire has been cut at the top end and etched at the bottom end to form the tip. One can estimate its height as that of an effective cylinder, as indicated in the picture, *h* = (574 ± 30) μm. However, this quantity is only representative of this particular qPlus sensor and is expected to vary from one sensor to another. The free prong has a width *w* = (132 ± 7) μm and a thickness *t* = (222 ± 12) μm, as measured from Figure 1c, which is the magnification of the dotted rectangle shown in Figure 1b. From these geometric quantities, the estimated static stiffness of the probe (cf. Equation 2) is *k*_s_ = (3322 ± 1270) N/m. To get this value, we have considered Young’s modulus and density of quartz, *E* = (78.7 ± 1.6) GPa and ρ = (2.65 ± 0.06) × 10^3^ kg·m^−3^, respectively. It is also reminded that Equation 2 does not include the contribution of the tip.

The qPlus sensor is assumed to be in thermal equilibrium at *T* = 9.8 K. The temperature is measured within the head of the microscope by a Si diode and readout by a Lakeshore 335 Controller. The microscope being in closed-cycle has been thermalized at that temperature for several weeks. A HQA-15M-10T charge preamplifier from Femto collects the piezoelectric current generated by the qPlus sensor [28] and sends the preamplified signal into a Nanonis OC4 oscillation control unit for digital conversion and a Nanonis Mimea SPM control system from SPECS for processing this signal. An analog low-pass filter in the OC4 acts as an antialiasing filter before digital conversion by an ADC. It is implemented as a third-order overall Butterworth filter with a Sallen–Key topology and with a fixed cut-off frequency of 5 MHz, meaning that dampening is 60 dB per decade. There is no additional filter in the analog part, and there is no antialiasing filter in the digital domain. Before thermal noise measurements, the qPlus deflections are accurately calibrated into metric units with a custom-made script implemented in the Nanonis MIMEA control unit that performs the constant-γ calibration procedure of the oscillation amplitude in nc-AFM mode [16–18]. The accuracy of the calibration is cross-checked in STM mode, which guarantees an accuracy of 5% in the amplitude calibration.

A dedicated software data acquisition module implemented by SPECS into the Nanonis MIMEA control software is used to acquire the *M*_exp_ time traces of the signal *z*(*t*) featuring the thermal fluctuations of the qPlus sensor and to process them accordingly to yield the averaged rms PSD spectrum of the thermal fluctuations (cf. subsection “Acquisition parameters of the thermal fluctuations”).

Because *M*_exp_ ≃ 500 time traces are acquired, lasting *T*_w_ ≃ 50 s each (cf. also subsection “Acquisition parameters of the thermal fluctuations”), the total acquisition lasts a couple of hours. In order to lower the parasitic noise level, these measurements are carried out overnight. The protocol might seem long and demanding, but it is the properties of the qPlus sensor that constrain one to drastic acquisition parameters. In 2013, Lübbe et al. had already come to the same conclusion in the case of silicon cantilevers in UHV at room temperature [56]. To overcome the drawback of the acquisition duration with high-*Q* probes, they introduced a quick and efficient alternative method based on the spectral analysis of the frequency shift detected by the PLL. If their concept was transposable to the case of qPlus sensors without loss of accuracy in the calibration, which is not established so far, it would be an advantage over the current method.

During the thermal noise measurement (*S_z_*(*f*)), the qPlus sensor is located far from the sample such that no interaction force may develop between tip and surface. All inputs to the microscope are grounded (e.g., high voltage lines of the *X*, *Y*, and *Z* scanner, coarse motor, and bias). The tunneling current readout is also grounded. It is also made sure that no parasitic external noise source (mechanical or electrical) adds to the measurement. The measurement noise PSD (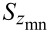
(*f*)) is recorded under similar conditions, except that the input of the charge amplifier is not connected and let open (qPlus sensor not connected). The acquisition parameters of *S_z_*(*f*) and 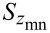
(*f*) are detailed hereafter.

### The qPlus sensor seen as an equivalent SHO

As with many other results dealing with that topic, a central assumption of this work is that the mechanical behavior of the qPlus sensor may be described by that of an equivalent SHO of resonance frequency *f*_1_, quality factor *Q*_1_, amplitude at the resonance *A*_1_, and stiffness *k*_1_. The relevance of that approximation is verified by recording the resonance curve around *f*_1_ and checking to which extent the measured amplitude *A*(*f*) and phase φ(*f*) can be fitted by the SHO model for large quality factors (cf. section “Experimental results”, subsection “Equivalent SHO”):


[19]

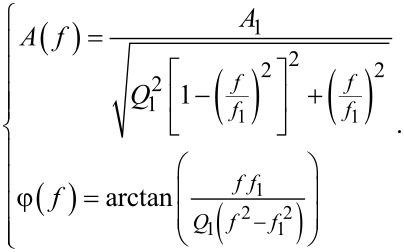



Doing so, *f*_1_ and *Q*_1_ will be determined accurately. For the sake of the forthcoming discussions, we use typical orders of magnitude for qPlus sensors operated in LT UHV, namely *f*_1_ ≃ 25 kHz and *Q*_1_ ≃ 2 × 10^5^.

### Acquisition parameters of the thermal fluctuations as a discrete time signal

The concept of PSD applied to the measurement of thermal fluctuations was introduced by assuming a continuous, that is, analog time signal. But on the experimental level, the thermal fluctuations are meant to be processed by the Nanonis MIMEA control unit, which is based on a digital FPGA architecture, such that the signal is ultimately discrete in time and of finite duration *T*_w_. Let us assume the signal to be sampled with a period *T*_s_, that is, a sampling frequency *f*_s_ = 1/*T*_s_, yielding a buffer of *N* samples. The windowing duration is then:


[20] $$T_{\text{w}}=NT_{\text{s}}=\frac{N}{f_{\text{s}}}.$$

In UHV and at low temperature, the qPlus sensor’s thermal fluctuations stem from stochastic phonons of the quartz in thermal equilibrium with the thermostat at the energy *k*_B_*T*. The phonons excite the qPlus sensor and statistically repeat over time with a frequency spectrum yielding a thermal noise rms PSD described by that of an equivalent SHO (Equation 17). The spectrum conceals the mechanical properties of the SHO and exhibits a resonance at *f*_1_. Thus, stochastic phonons with frequencies at, or close to, the SHO resonance frequency, produce long-standing oscillations, particularly if the SHO’s quality factor is large, which is the case with qPlus sensors. It is therefore mandatory to acquire the thermal fluctuations over a duration window *T*_w_ that is much larger than the intrinsic equilibration time of the SHO defined as τ_1_ = 2*Q*_1_/*f*_1_, hence, *T*_w_ ≫ τ_1_. With the orders of magnitude that were chosen above, τ_1_ ≃ 16 s.

Furthermore, setting *T*_w_ implicitly means setting δ*f*, that is, the frequency resolution δ*f* of the rms PSD spectrum:


[21] $$\delta f=\frac{f_{\text{s}}}{N}=\frac{1}{NT_{\text{s}}}=\frac{1}{T_{\text{w}}}.$$

Therefore, the problem is to determine a correct value for δ*f* that must satisfy δ*f* ≪ 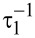. With our parameters, δ*f* ≪ 60 mHz. We arbitrarily set δ*f* = 20 mHz, that is, *T*_w_ = 50 s. According to the definition of the quality factor, the SHO bandwidth is *w_f_* = *f*_1_/*Q*_1_, that is, here *w_f_* ≃ 125 mHz. Thus, it is essential to have at least ≃7 samples within the *w_f_* bandwidth around *f*_1_ in the tn-PSD.

On the hardware level, the analog signal of the qPlus deflections is sampled at the maximum rate imposed by the MIMEA control unit, *f*_s_*_,_*_max_ = 40 MHz, resulting in a Shannon–Nyquist frequency of 20 MHz. At that frequency, because of the analog antialiasing filter, the digital signal provided by the ADC is dampened by 36 dB. However, *f*_s_*_,_*_max_ is not the sampling frequency used to perform the thermal noise analysis, as *T*_w_ ≃ 50 s would imply a too large buffer of samples to handle (*N* = *T*_w_*f*_s_*_,_*_max_ ≃ 2 × 10^9^). Therefore the discrete time signal is downsampled at *f*_s_ while making sure that *f*_s_ fulfills the Shannon–Nyquist sampling theorem *f*_s_ *>* 2*f*_1_. To limit memory usage on the hardware, the maximum buffer size is limited to *N* = 8,388,544 samples. Thus, because *f*_1_ ≃ 25 kHz, we set *f*_s_ = 156.250 kHz (*T*_w_ = *N*/*f*_s_ ≃ 53.6 s). This results in an acquisition bandwidth of the thermal fluctuations of *B*_s_ = *f*_s_/2 = 78.125 kHz. Note that, because the second flexural eigenmode features a resonance frequency *f*_2_ ≃ 6.27 *f*_1_ ≃ 156.750 kHz *> f*_s_/2, the corresponding discrete time signal will be downsampled and, hence, not properly detected. Therefore, our framework to the stiffness calibration restricts the detection bandwidth of the qPlus sensor’s thermal fluctuations to its fundamental eigenmode. It is reminded that no additional digital filtering that might act as an antialiasing filter is used for the acquisition of the thermal noise (cf. section “Numerical Simulations”, subsection “Ideal case: no measurement noise”).

To summarize, for qPlus sensors operated in LT UHV, typical sampling parameters of the discrete time signal of the thermal fluctuations are: *T*_w_ ≃ 53 s, *f*_s_ = 156.250 kHz, *T*_s_ = 1/*f*_s_ = 6.4 μs, δ*f* ≃ 19 mHz, yielding a buffer of *N* ≃ 8.4 × 10^6^ samples.

## Numerical Simulations

The simulation of AFM experiments affected by stochastic noise and its consequences on the statistics of the PSD has been addressed by Labuda and coworkers [85–86]. Specifically, the authors use inverse Fourier transform for generating time-domain stochastic noise directly from a numerically defined PSD of stationary noise. The method is valuable as the defined PSD may, for example, be fitted from an experimental measurement.

Our approach is the opposite. We simulate the time trace of the thermal fluctuations and derive the tn-PSD consistently via direct discrete Fourier Transform. Our framework restricts the experimental detection bandwidth of the qPlus sensor’s thermal fluctuations to its fundamental eigenmode. However, the numerical description of the qPlus sensor’s mechanical behavior must be as accurate as possible. As already mentioned, the Euler–Bernoulli model accounts for the probe deflection from the superposition of the deflections of each of its eigenmodes described as independent SHOs. With our computational means however, it is unrealistic to describe a too large number of eigenmodes presenting high-*Q* factors. To gain computational time, we restrict the numerical analysis to the first three eigenmodes of the qPlus sensor (*n* = 1–3). Furthermore, we do not account for the influence of the tip (*m*_tip_ = 0). The as-calculated values of the set (*f_n_*, *Q_n_*, *k_n_*) are reported in Table 1. We chose for (*f*_1_, *Q*_1_, *k*_1_) the values (25 kHz, 10^5^, 1800 N/m). The values of *f*_2_ (*f*_3_) and *k*_2_ (*k*_3_) are given by Equations S9 and S10 of Supporting Information File 1, respectively, with α*_n_* given by Equation 5. As for *Q*_2_ and *Q*_3_, there are few results in the literature on how they are expected to vary in UHV. Usually, the quality factor decreases with *n* (cf. [87] and silicon cantilever P5 in [56]). We therefore have set arbitrary values following that trend. The simulated temperature is 9.8 K.

**Table 1:** Values of *f_n_*, *Q_n_*, and *k_n_* for the first three eigenmodes of the simulated qPlus sensor. We only set the values of the fundamental eigenmode (*n* = 1), the others are derived from the corresponding equations.

Eigenmode	α*_n_* ()	*f_n_* (kHz)	*Q_n_* ()	*k_n_* (N/m)
(*n*)	(Equation 5)	(cf. Supporting Information File 1, Equation S9)		(cf. Supporting Information File 1, Equation S10)

**1**	1.875	25.000	10^5^	1800
**2**	4.694	156.680	50 × 10^3^	70 703
**3**	7.864	438.650	25 × 10^3^	554 150

We first consider the ideal case where there is no measurement noise and then introduce measurement noise, whose characteristics reproduce those of the experimental noise.

### Ideal case: no measurement noise

Assuming *F*_th_*_,n_*(*t*) to be the instantaneous value of the thermal force applied to the qPlus sensor’s *n*-th eigenmode, the corresponding instantaneous deflection *z*_th_*_,n_*(*t*) obeys the classical second-order differential equation for the SHO:


[22]

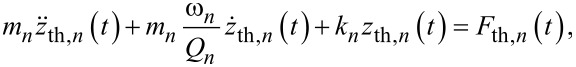



where ω*_n_* = 2π*f_n_* and *m_n_* = 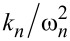 are the resonance angular frequency and mass of the equivalent SHO representing the *n*-th eigenmode, respectively.

The thermal noise is simulated as stemming from a random stationary process, a reasonable assumption for thermal noise. Then, the FDT states that the thermal force inducing thermal fluctuations of each eigenmode is normally distributed with a rms standard deviation given by (cf. Supporting Information File 3, Equation S11):


[23] $$F_{\text{th},n}\left(t\right)=\sqrt{\frac{2k_{\text{B}}Tk_{n}}{\pi Q_{n}f_{n}}B_{\text{s,max}}},$$

where *B*_s_*_,_*_max_ = *f*_s_*_,_*_max_/2 is the acquisition bandwidth of *z*_th_*_,n_*(*t*). It is important to notice that *B*_s_*_,_*_max_ is not to be confused with *B*_s_, which was described as the experimental acquisition bandwidth of the thermal fluctuations. Here, *B*_s_*_,_*_max_ is the acquisition bandwidth of the numerical signal *z*_th_*_,n_*(*t*), which describes the instantaneous deflection of the qPlus sensor, that is, a continuous time signal, whose experimental counterpart is sampled by the control unit at a maximum rate *f*_s_*_,_*_max_ = 40 MHz, as already mentioned.

With our computational means it is not possible to account for such a large sampling frequency. We, therefore, have restricted it to an integer multiple of *f*_s_ = 156.250 kHz, namely 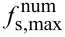 = 20 × *f*_s_ = 3.125 MHz. Thus, Equation 22 is integrated with a Runge–Kutta 4 scheme by using a sampling period 
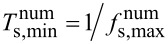
 = 320 ns, embodying the discrete time signal processed by the control unit for thermal noise analysis. We have noticed that the relevance of Runge–Kutta methods to solve stochastic differential equations seems debated [88–89]; however, at our level, this algorithm was found to be more accurate than the Symplectic Euler or the Verlet (Leapfrog) algorithms (data not shown).

Consistently with the estimated acquisition parameters, we simulate a window duration *T*_w_ = 60 s. Because the three SHOs are independent, the total deflection of the qPlus sensor is given by:


[24]

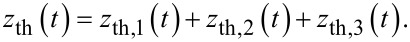



**Figure 2:**
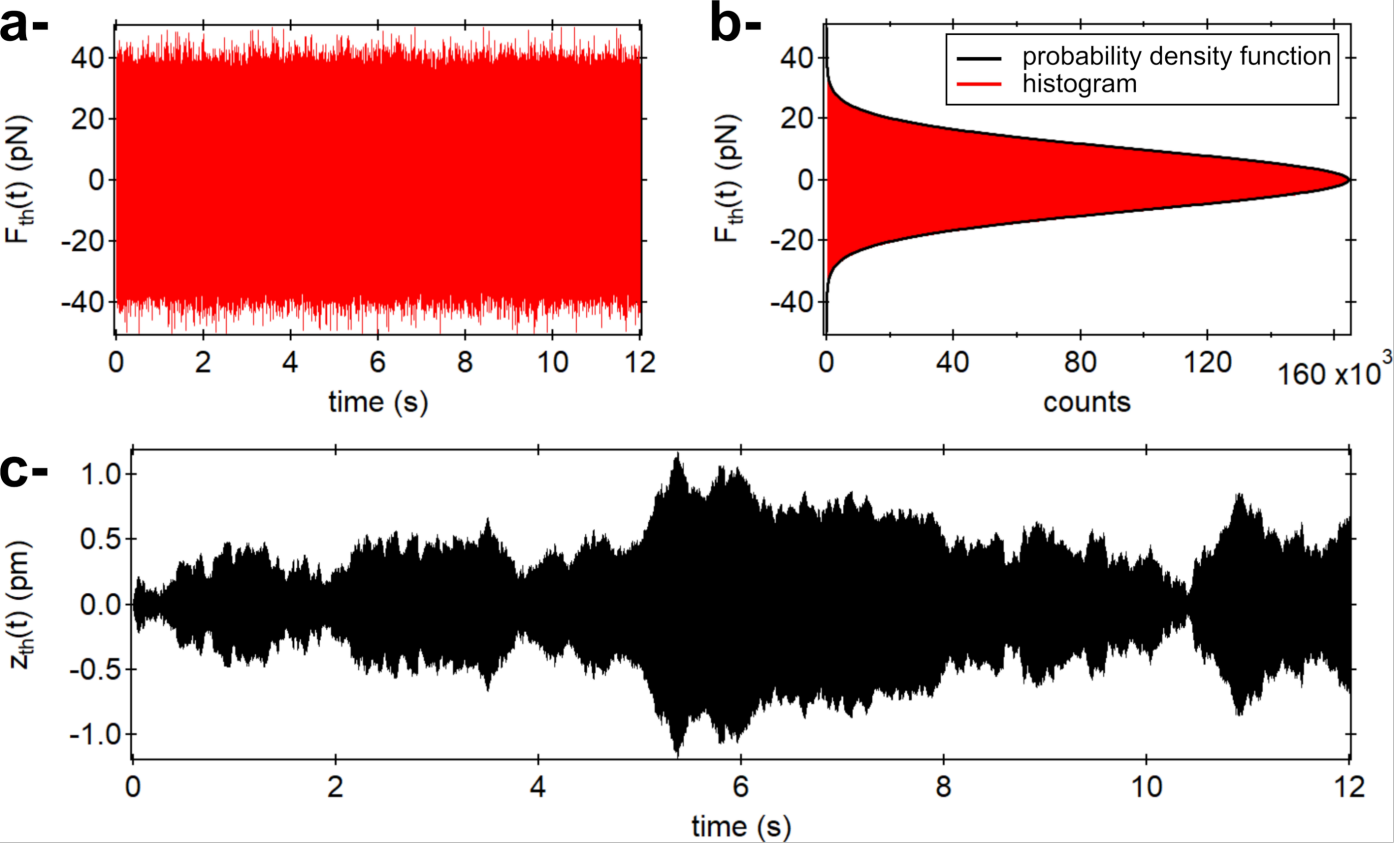
a- Time trace of the simulated thermal force for the first eigenmode displayed over an arbitrary duration of 12 s. b- Corresponding histogram and probability density function showing its normal distribution (thick black curve). c- Corresponding time trace of the fluctuations of the simulated qPlus (superposition of the first three eigenmodes, cf. text).

An example of a single simulated time trace of *z*_th_(*t*) is reported in Figure 2, along with the stochastic force (first eigenmode only). The thermal force histogram exhibits a normal distribution with a rms standard deviation given by Equation 23. The corresponding probability density function (cf. Supporting Information File 3, Equation S12) is shown in black.

A set of *M*_num_ = 64 time traces of *z*(*t*) is thus calculated. Then, each trace *z*(*t*) is downsampled with a rate *r* = *f*_s_*_,_*_max_/*f*_s_ = 20 to form a signal *z_d_*(*t*) of duration *T*_w_ = 60 s sampled at *f*_s_ = 156.250 kHz. This results in a thermal noise acquisition bandwidth *B*_s_ = *f*_s_/2 = 78.125 kHz, consistent with the experimental conditions.

**Figure 3:**
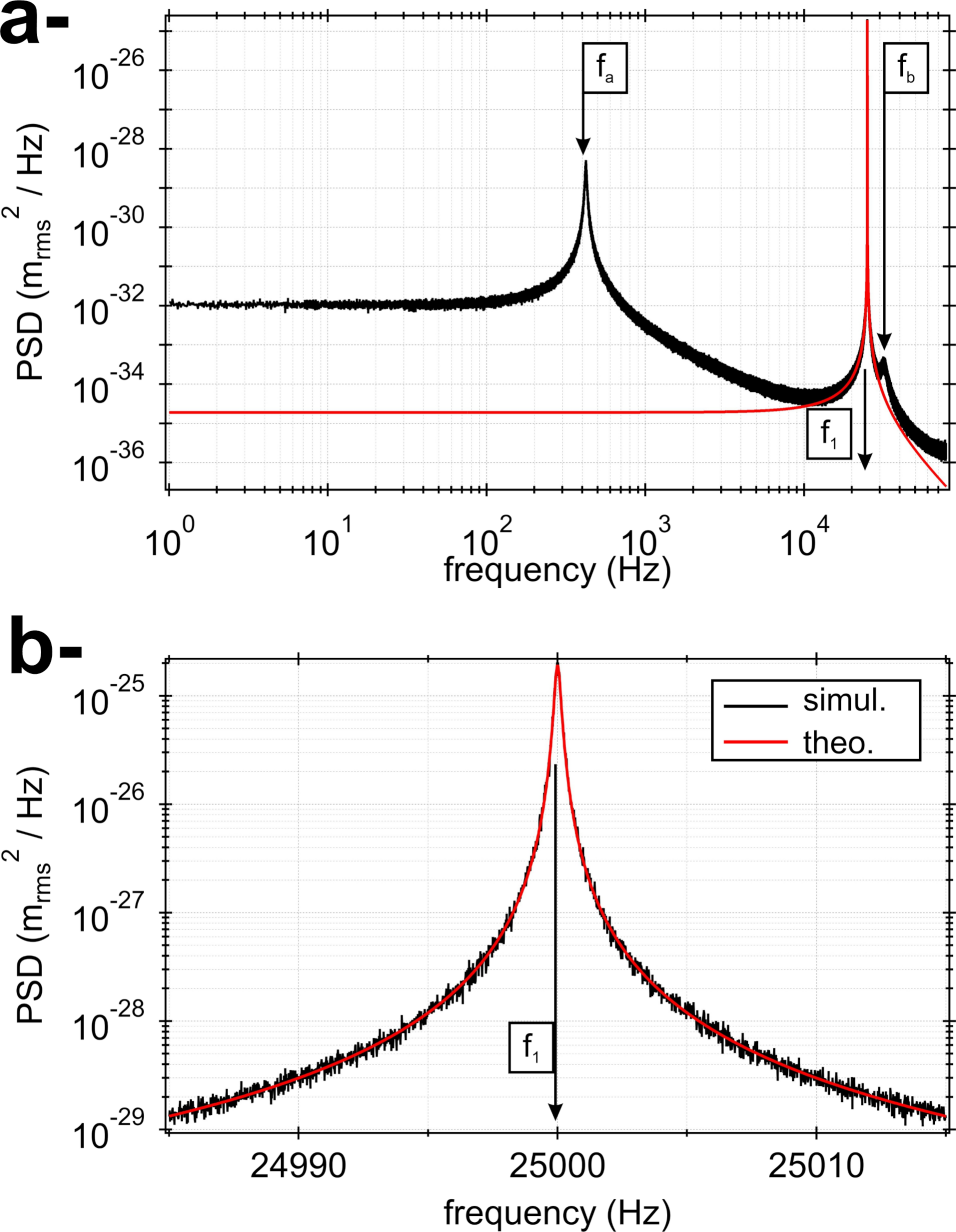
a- Simulated tn-PSD spectrum at 9.8 K (spectral range [0;*B*_s_]) for the qPlus sensor whose mechanical parameters are given in Table 1 (black curve). The resonance peak due to the fundamental eigenmode (*f*_1_ = 25.000 kHz) largely dominates the tn-PSD. The theoretical tn-PSD for the SHO is displayed in red. Additional peaks (*f*_a_ and *f*_b_) stemming from eigenmodes 2 and 3 are visible because of spectral aliasing, which also explains the discrepancy with respect to the SHO tn-PSD (cf. text). b- tn-PSD displayed in the spectral range ±15 Hz around *f*_1_ (simulated data: black curve, SHO tn-PSD: red curve).

A typical spectrum is reported in Figure 3a in the spectral range [0;*B*_s_] (black curve). A zoom around *f*_1_ = 25 kHz (spectral range [24.987 kHz; 25.013 kHz]) is shown in Figure 3b. The curve exhibits a pronounced resonance, representing the resonance frequency of the first eigenmode of the simulated qPlus sensor with *Q*_1_ = 10^5^. The continuous red curve is the theoretical tn-PSD calculated with Equation 17. Around the resonance, the agreement between both curves is excellent. Deriving the stiffness according to method 1 (PSD integral over the range [0;*B*_s_], Equation 15) gives 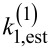 = 1750 N/m. Method 2, using Equation 17 as fit functional, gives 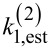 = 1765 N/m, while method 3 gives 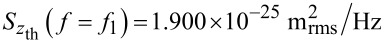, that is, 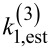 = 1813 N/m. The results are gathered in Table 2. The relative error is less than 3% with respect to the nominal value *k*_1_ = 1800 N/m, regardless of the method.

**Table 2:** Calibrated stiffness using methods 1, 2 or 3. The number of averaged time traces is *M*_num_ = 64 in each case. The estimated stiffness is in excellent agreement with the nominal value.

Method	Nominal *k*_1_	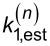
(*n*)	(N/m)	(N/m) (rel. err.)

**1**	1800	1750 (−2.8%)
**2**	1800	1765 (−1.9%)
**3**	1800	1813 (+0.7%)

In addition to the resonance peak at *f*_1_, the black curve in Figure 3a exhibits two additional unexpected peaks, resembling resonance peaks, at *f*_a_ ≃ 433 Hz and *f*_b_ ≃ 31.7 kHz. However, their magnitudes are by several decades smaller than that of the *f*_1_ peak. These peaks are aliases of the eigenmodes 2 and 3 of the qPlus sensor due to spectral aliasing within the band [0;*B*_s_]. The spectral aliasing is also responsible for the discrepancy between the simulated tn-PSD and the theoretical tn-PSD of the SHO in the low-frequency part of the spectrum, which is discussed in Supporting Information File 4, along with the influence of an additional digital antialiasing filter.

### Influence of measurement noise

The experimental data are subject to measurement noise. To assess how it might influence the stiffness calibration, two types of measurement noise sources that embody the experimental measurement noise are introduced in the simulations. The first one is white noise featuring a constant rms PSD spectrum, 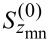. The second source has a 1/*f^n^* rms PSD spectrum, 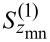
(*f*). For *n* = 1, one has a regular 1/*f* noise. The discussion is carried out with the theoretical tn-PSD of the SHO (Equation 17), and a similar acquisition bandwidth *B*_s_ = 78.125 kHz is considered. Then, the total rms PSD of the system is written as:


[25] $$S_{z}\left(f\right)=S_{\text{SHO}}\left(f\right)+S_{z_{\text{mn}}}\left(f\right),$$

where:


[26]

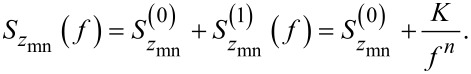



To make the discussion relevant, the numerical parameters defining 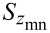
(*f*) are those deduced empirically from the experimental PSD (cf. section “Experimental results” and Figure 6), namely:


[27] $$S_{z_{\text{mn}}}\left(f\right)=3.765\times 10^{-27}+\frac{1.1\times 10^{-20}}{f^{1.6}}.$$

**Figure 4:**
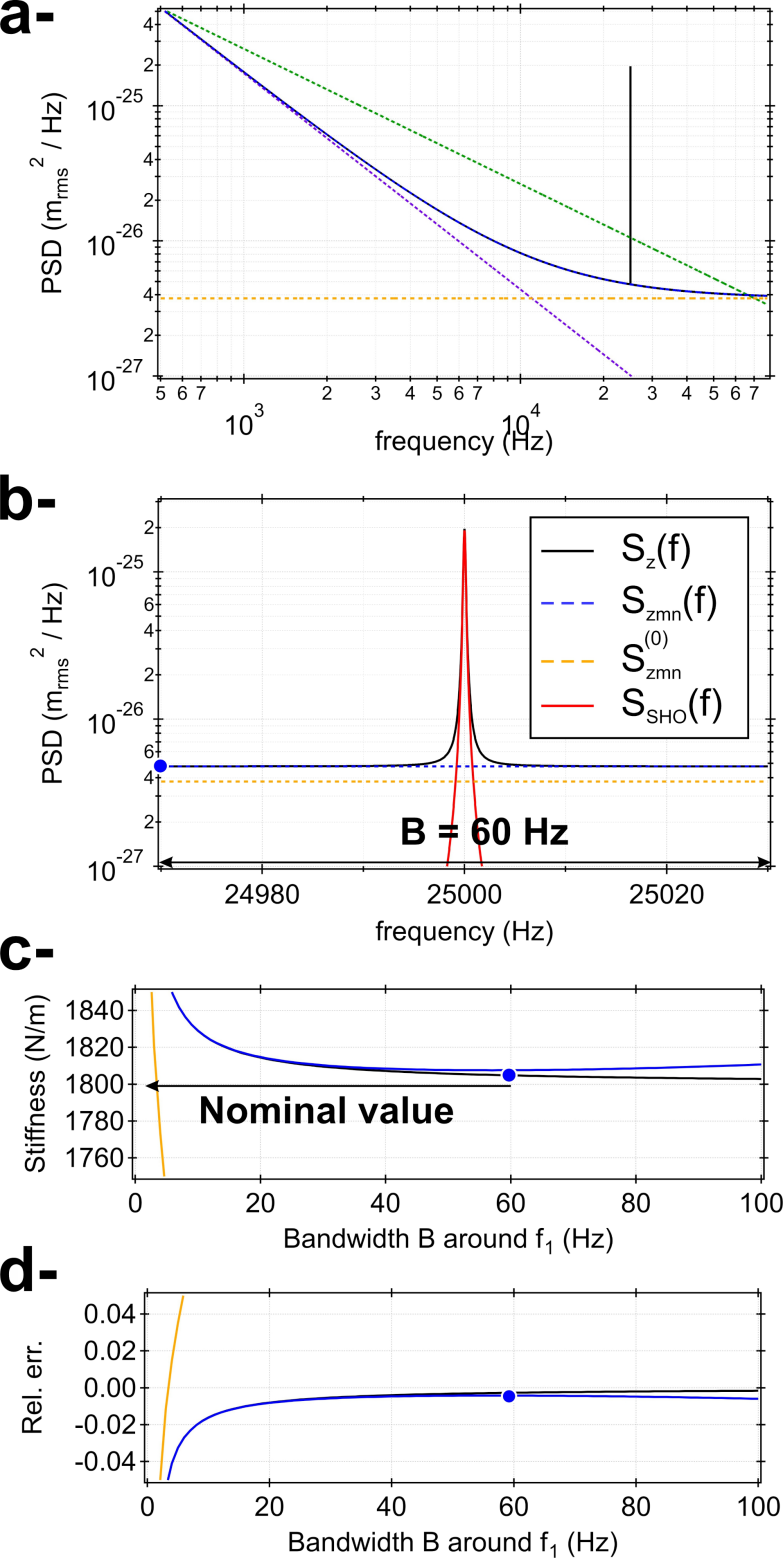
a- Numerical tn-PSD including measurement noise displayed in the range [500 Hz;*B*_s_]: *S_z_*(*f*) = *S*_SHO_(*f*) + 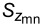
(*f*) (black curve). The measurement noise PSD, 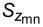
(*f*), is displayed as a dotted blue curve on top of *S_z_*(*f*), except at the resonance. The numerical parameters are given in the text. 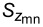
(*f*) is built as the superposition of a constant background 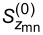 (dotted orange curve) and a 1/*f^n^*-like contribution 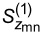
(*f*) = *K*/*f^n^* (dotted purple curve). Here, *n* = 1.6 to match with the experimental PSD (cf. text). A 1/*f* component has been displayed as a guide to the eye (dotted green curve). b- Zoom in the spectral range ±*B*/2 = ±30 Hz around *f*_1_. The theoretical PSD of the equivalent SHO is overlaid (red curve). In that area of the PSD, the noise floor may be estimated from the value of 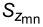
(*f*_1_ − *B*/2) (blue circle). c- Estimated stiffness upon integration of (i) *S_z_*(*f*) − 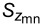
(*f*) (black curve), (ii) *S_z_*(*f*) − 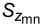
(*f*_1_ − *B*/2) (blue curve), and (iii) *S_z_*(*f*) − 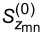 (orange curve), for an increasing bandwidth around *f*_1_. The nominal stiffness is *k*_1_ = 1800 N/m. d- Corresponding relative errors.

As for *S*_SHO_(*f*), (*f*_1_, *Q*_1_, *k*_1_) = (25 kHz, 10^5^, 1800 N/m). The corresponding spectrum is reported in Figure 4a.

According to method 1, we first make sure that


[28]

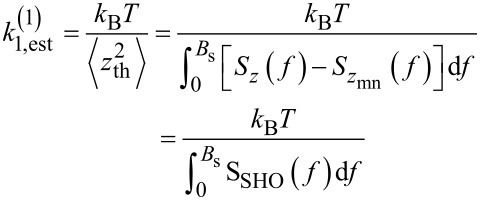



accurately leads to the nominal value 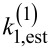 = 1800 N/m.

Practically, the measurement noise PSD may be difficult to account for with a functional as simple as Equation 27. This is why, in the literature, the stiffness may also be found to be estimated by integration of the PSD within a restricted bandwidth around the resonance [48], where its behavior is easier to describe. In the mentioned reference, however, the authors state that they compensate for the finite integration range, but without detailing how. We hereafter propose an analysis of the influence of a finite PSD integration range on the stiffness calibration.

In a ±30 Hz wide area around *f*_1_, the PSD noise background looks flat (cf. Figure 4b), as if the measurement noise only consisted of a constant, that is, white background noise. This effect essentially stems from the high *Q* value of the qPlus sensor, which imposes a weak bandwidth *w_f_* = *f*_1_/*Q*_1_ = 125 mHz around *f*_1_, over which the 1/*f^n^* attenuation, with our parameters, is barely visible. To assess how much the estimated stiffness is sensitive to the PSD integration interval, we derive the stiffness upon integration of the quantity *S_z_*(*f*) − 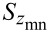
(*f*) over an increasing bandwidth *B* around *f*_1_ according to:


[29]

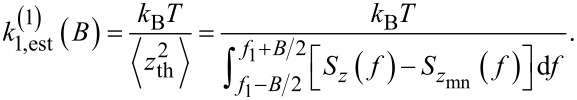



The result is reported in Figure 4c (black curve). The estimated stiffness fits to better than 2% with the nominal value as soon as the integration bandwidth is larger than *B*/2 = 5 Hz (cf. Figure 4d, black curve).

Then, we assess how much the estimated stiffness is falsified if only a constant background is subtracted from *S_z_*(*f*) instead of 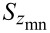
(*f*), regardless of the PSD power-law dependence at low frequencies. Following the same methodology, the estimated stiffness is derived for two cases, namely by integration of (i) *S_z_*(*f*) − 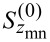 (removal of the white noise only, cf. orange curve in Figure 4c), and (ii) 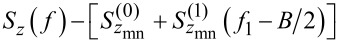 (removal of the white noise and the 1/*f^n^* noise, but the former is estimated at *f* = *f*_1_ − *B*/2; here 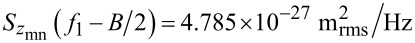, cf. blue curve and blue circle in Figure 4c). The accuracy of the estimated stiffness remains excellent by integration of the quantity 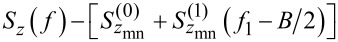 and nearly equal to that obtained upon integration of *S_z_*(*f*) − 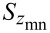
(*f*) (black vs blue curve in Figure 4c) up to *B*/2 = 30 Hz. Conversely, 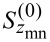 underestimates the background noise in the area around *f*_1_ such that the integration of the quantity *S_z_*(*f*) − 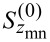 yields a largely underestimated stiffness value (orange curves in Figure 4c,d).

In summary, this section has proven that the stiffness of a qPlus sensors may be accurately calibrated by integration of the tn-PSD in a restricted bandwidth *B* around *f*_1_, provided that (i) *S_z_*(*f*) presents a homogeneous background measurement noise without parasitic peaks within *B*, (ii) *B* is much larger than the equivalent SHO bandwidth *w_f_*, and (iii) *S_z_*(*f*) is corrected from the measurement noise, whose spectral dependence has been estimated over *B* or corrected from a constant measurement noise estimated nearby *f*_1_; however, depending on how the constant background is determined, that former approach is less accurate.

## Experimental Results

The experimental stiffness calibration was performed with two distinct qPlus sensors, referred to as qPlus 1 and 2 in the following. For qPlus 1, the tip was removed so as to reproduce the behavior of a perfectly free prong, which allows us to benchmark the thermal noise measurement method on two probes with expected distinct mechanical behaviors.

### Equivalent SHO

**Figure 5:**
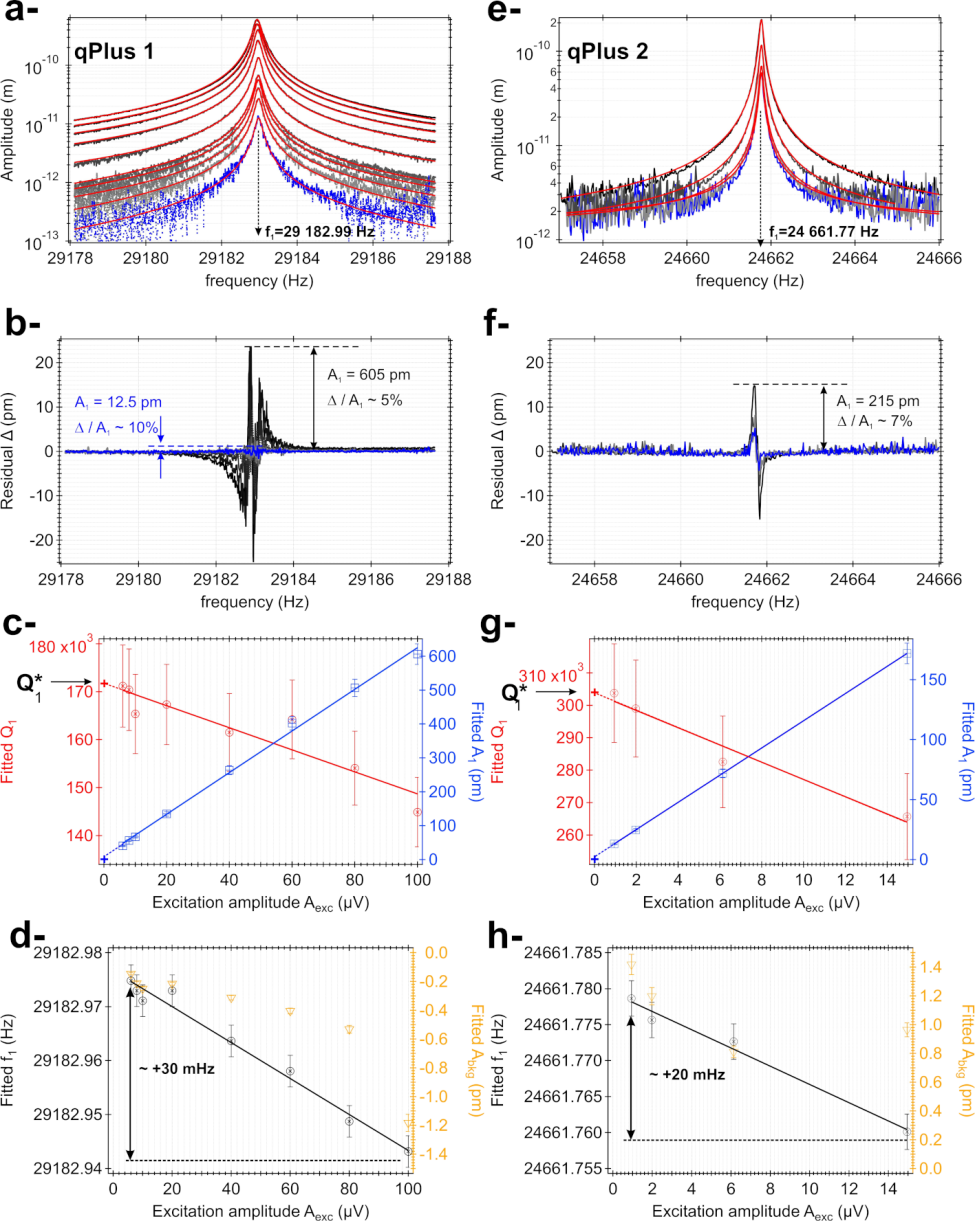
Experimental resonance curves of the fundamental eigenmode for two distinct qPlus sensors recorded with decreasing excitation amplitudes *A*_exc_ (a- and e-). For each curve, a fit with the SHO equations gives the values of resonance frequency *f*_1_, resonance amplitude *A*_1_, background *A*_bkg_, and quality factor *Q*_1_ (red curves). The curves acquired with the smallest excitation amplitude are shown in blue to discriminate them from the less noisy data. The residual Δ of each fit is shown in panels b- and f-. The relative magnitude of the ratio Δ/*A*_1_ at the resonance is between 5% and 10%. The evolution of *A*_1_ and *Q*_1_ vs *A*_exc_ is reported in panels c- and g- (right and left axes, blue and red markers, respectively). The evolution of *A*_bkg_ and *f*_1_ vs *A*_exc_ is reported in panels d- and h- (right and left axes, orange and black markers, respectively). The evolution of the fitted quality factor allows us to extract the intrinsic quality factor of each qPlus sensor, 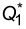 (cf. text).

In order to assess whether the mechanical behavior of the qPlus sensor reasonably compares to that of a SHO, several resonance curves (fundamental eigenmode) were measured for both qPlus sensors with decreasing excitation amplitudes (cf. Figure 5). Each curve is fitted with Equation 19 (amplitude dependence only), from which the resonance frequency *f*_1_, the resonance amplitude *A*_1_, and the quality factor *Q*_1_ are deduced. A constant background, *A*_bkg_, has been added to Equation 19 to account for the noise floor. A set of four coefficients is therefore ultimately used for the fits, namely *A*_1_, *Q*_1_, *f*_1_, and *A*_bkg_. The fits are performed by means of the software Igor Pro from Wavemetrics^®^. For the non-linear least-squares fit process used here, Igor uses the Levenberg–Marquardt algorithm looking for the minimum value of chi-squared. The confidence interval for the fit coefficients is set to 99%. The residual of each fit, Δ, is built according to Δ = *A*_exp_(*f*) − *A*_fit_(*f*). Doing so, we not only want to measure these parameters while estimating the fit quality, but also want to verify whether the qPlus quality factor is independent from the excitation amplitude (*A*_exc_), as expected in the SHO model. Since the thermal noise measurements are performed without mechanical excitation of the qPlus sensor and its quality factor is required to perform the stiffness calibration (methods 2 and 3), this value must reflect an intrinsic property of the probe. We name that quantity 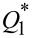. The quality factor used to perform the stiffness calibration must be as close as possible to 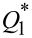, meaning that it must have been determined without any influence of the mechanical transfer function of the qPlus sensor holder within the microscope, that is, at extremely low excitation amplitude.

A set of excitation amplitudes yielding *A*_1_ values ranging from ≃500 pm down to ≃10 pm has been selected. The resonance curves are reported in Figure 5a and Figure 5e (qPlus 1 and 2, respectively). Each curve is acquired over an interval of ±4.8 Hz around the resonance (qPlus 1: *f*_1_ = 29,182.99 Hz, qPlus 2: *f*_1_ = 24,661.76 Hz) and features a spectral resolution equal to, or better than, the required one, δ*f* = 20 mHz. Each curve is acquired within a one hour time lap because of the large quality factor, which is enough to prevent any non-adiabatic effect from occurring. The fits with Equation 19 as fit functional are overlaid on each acquisition (red curves). The residuals are reported in Figure 5b and Figure 5f (qPlus 1 and 2, respectively).

Figure 5b,f shows that the magnitude of the residuals increases when *A*_exc_ is decreased owing to the degraded signal-to-noise ratio. Overall, in the vicinity of the resonance, the relative magnitude increases from less than 5% (*A*_1_ = 605 pm) to less than 10% (*A*_1_ = 12.5 pm). Although the following statement is not rigorous, we estimate that a relative uncertainty of ±5% is quite representative for the fit coefficients (except *f*_1_, which is estimated with a much better accuracy; cf. below). They are plotted consistently, but note that these error bars are much larger than the standard deviations given by the fit process for each coefficient. We have noticed that some approaches are reported to achieve specified tolerances on the fit coefficients of spectra exhibiting a Lorentzian response; however, we did not employ them here [53,90].

The evolution of the fitted quality factor as a function of *A*_exc_ for qPlus 1 and 2 is reported in Figure 5c and Figure 5g, respectively (red symbols and continuous red line, left axis). The evolution of the fitted value of *A*_1_ as a function of *A*_exc_ is reported as well (blue symbols and continuous blue line, right axis). For *A*_1_ ≃ 100 pm, the quality factor of qPlus 1 is in the range of 168,000, whereas for qPlus 2, it is rather 280,000. The correct expected linear dependence between *A*_1_ and *A*_exc_ is observed down to *A*_exc_ = 0 (dotted line and blue cross), which testifies that, with these excitations, non-linearities due to the mechanical transfer function of the qPlus excitation system are not likely to occur.

The evolution of the fitted value of the resonance frequency as a function of *A*_exc_ for qPlus 1 and 2 is reported in Figure 5d and Figure 5h, respectively (black symbols and continuous black line, left axis). The evolution of the fitted value of *A*_bkg_ as a function of *A*_exc_ is reported as well (orange symbols and orange curve, right axis). Because of the high quality factors, the fits yield values of *f*_1_ with a high accuracy, such that it is irrelevant to plot the fitted value for that coefficient with 5% error bars. We rather use the standard deviation given by the fit, which is about ±*f*_1_ × 10^−7^ ≃ ±3 mHz. We note that the fitted values for *A*_bkg_ remain consistently small compared to *A*_1_ and that a significant offset error in the estimation of the amplitude is unlikely to occur.

For both qPlus sensors, the quality factor and the resonance frequency feature a moderate linear dependence with *A*_exc_. Both *Q*_1_ and *f*_1_ increase as *A*_exc_ decreases. *Q*_1_ increases by about 20% (140,000→170,000 for qPlus 1, 260,000→310,000 for qPlus 2) and *f*_1_ by about +30 mHz. A similar effect on *f*_1_, with comparable magnitudes, has been reported by Dagdeviren et al. with other types of qPlus sensors [91]. The reference states that the drop of the resonance frequency upon increase of the oscillation amplitude results from an in-plane surface stress near the clamp of the free prong of the QTF. In the mentioned reference, the evolution of the quality factor is not reported. Hence, it is difficult to conclude whether the in-plane surface stress is the main cause for the observed trend of the quality factor. Thus, even though the observations by Dagdeviren et al. are consistent with ours, we do not exclude the additional influence of the mechanical transfer function of the excitation system onto the QTF. Indeed, it is known that the way the probes are mounted on their holders, along with the mechanical transfer function of the probe excitation system, may influence their quality factor [92], which is detrimental to the decoupling of conservative and dissipative forces in nc-AFM [93]. Because much less mechanical energy is injected into the system when *A*_exc_ is decreased, energy losses are reduced, and it is the most reactive part of the system, namely the QTF, that reacts to the excitation.

From these measurements, we deduce the 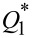 value of each qPlus sensor (extrapolation at *A*_exc_ = 0, dotted line and red cross). For qPlus 1 and 2, we get, respectively, 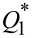 = 172,000 and 304,000. These values are used in the following to perform the stiffness calibration.

### Measurement noise PSD

**Figure 6:**
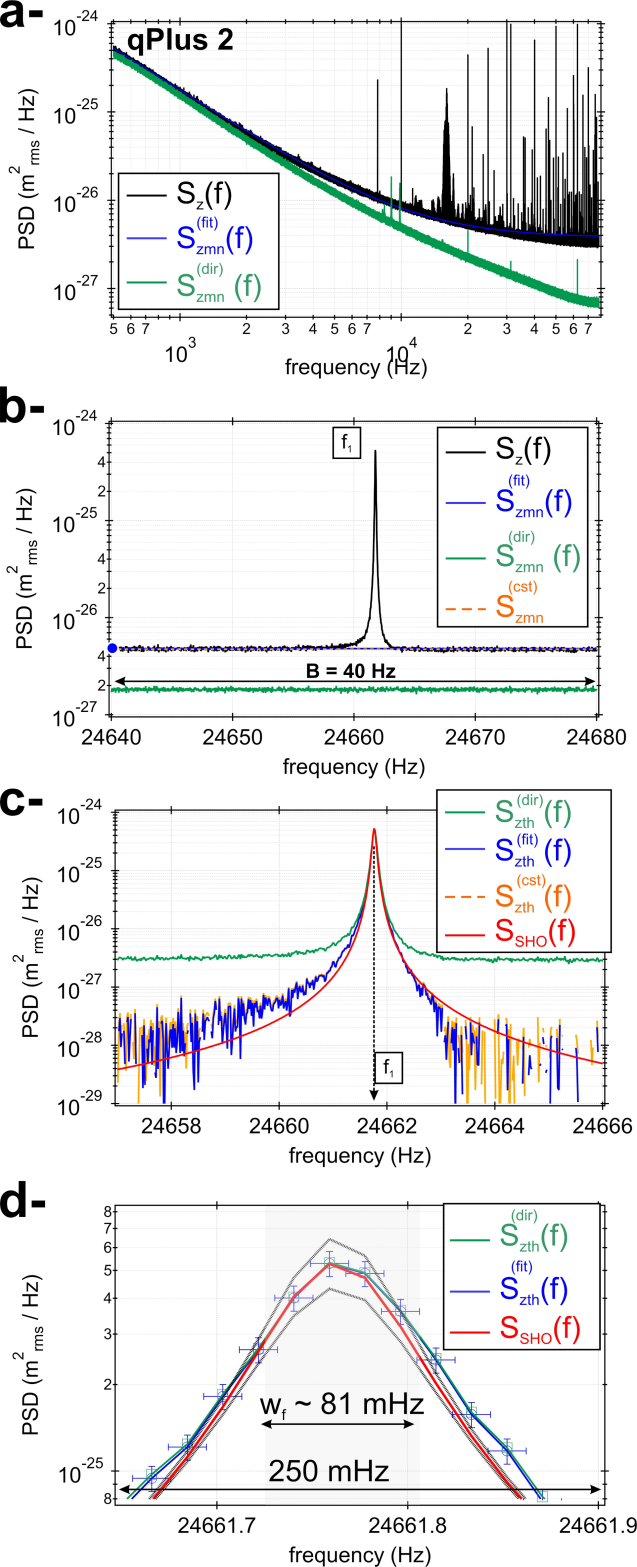
a- Experimental tn-PSD featuring measurement noise, *S_z_*(*f*), for qPlus 2 (black curve). The empirically derived measurement noise PSD 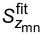
(*f*) is displayed in blue, whereas the directly measured homologous quantity 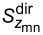
(*f*) is displayed in green (cf. text). b- Zoom area around the resonance of the qPlus sensor (40 Hz wide). The traces of 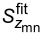
(*f*) and 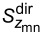
(*f*) are visible. The dotted orange curve depicts a constant noise background 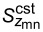 that coincides with 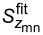
(*f*) in that spectral range. c- tn-PSD for qPlus 2 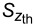
(*f*) derived according to (i) *S_z_*(*f*) − 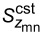 (dotted orange curve), (ii) *S_z_*(*f*) − 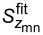
(*f*) (blue curve), and (iii) *S_z_*(*f*) − 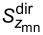
(*f*) (green curve). The estimated stiffness is derived from these measurements and gathered in Table 3. The red curve is the theoretical SHO PSD calculated from the experimentally deduced parameters of the qPlus sensor. d Zoom area on top of the resonance shown in panel c- (250 mHz wide). The bandwidth of the equivalent SHO, *w_f_* ≃ 81 mHz, has been overlaid in light grey. The experimental data are framed by the theoretical SHO PSD with an uncertainty smaller than 10% (cf. text).

Figure 6a reports the measured tn-PSD spectrum with measurement noise, *S_z_*(*f*), for qPlus 2 (black curve), for which we now have identified *f*_1_ = 24,661.76 Hz and 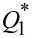 = 304,000. The spectrum was acquired with the parameters *f*_s_ = 156.250 kHz, *N* = 8,388,544 samples, and *M*_exp_ = 512 time traces. It is reported over the bandwidth [500 Hz;*B*_s_] since the qPlus charge preamplifier features a cut-off high-pass frequency of ≃500 Hz [28]. The spectrum exhibits a bunch of parasitic peaks for frequencies above 10 kHz. Owing to the large spectral resolution, extremely narrow ones can be identified, tracing the influence of purely sinusoidal noise components. Some others look wider. As mentioned earlier, one expects some of these to stem from aliasing effects, although this was not investigated in detail and neither was the origin of the purely sinusoidal noise components.

The magnified area of the spectrum around the qPlus resonance is reported in Figure 6b (40 Hz wide), where no parasitic peak is visible. The resonance is superimposed on a seemingly constant background measurement noise that is estimated to be 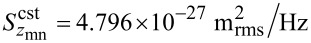 (orange dotted line in Figure 6b). 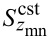 is the noise floor of the experimental setup in this frequency range. In Figure 6b, the experimental spectrum can qualitatively be compared with that of the numerical simulations (cf. Figure 4b). In the log–log scale, the spectrum features a linear decay from 500 to ≃5000 Hz (cf. Figure 6a). The estimated background measurement noise using Equation 26 as functional yields Equation 27. We name this quantity 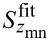
(*f*), although it was not deduced from a fit, but estimated empirically with the constraint to make it match 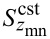 for *f* = *f*_1_ − 20 Hz (cf. blue dot in Figure 6b). The corresponding curve is reported in blue in Figure 6a,b. We notice that the charge preamplifier transfer function of the experimental setup induces a measurement noise drop (∝1/*f*^1.6^) that decays faster than regular 1/*f* noise.

The measurement noise PSD has also been estimated by direct measurement, that is, without connecting the qPlus sensor to the charge preamplifier input (green curve in Figure 6a,b). This third estimate of the measurement noise is named 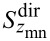
(*f*). For this acquisition, similar sampling parameters have been used. In the log–log scale, the spectrum exhibits a nearly linear decay all over the acquisition bandwidth. Some residual narrow noise components are still visible; but, overall, the spectrum is much cleaner than the tn-PSD one. The fact that both PSDs (qPlus connected and disconnected) are so different points towards an additional influence of noise when the qPlus sensor is connected. This is most probably due to the qPlus cabling inside the microscope; however, this issue was not addressed so far. It is, however, clear that 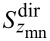
(*f*) will be insufficient to account for all the noise in the system and will, therefore, not be reliable to perform the stiffness calibration (cf. section below).

### Stiffness calibration

The tn-PSD 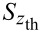
(*f*) of qPlus 2 deduced from 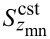, 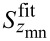
(*f*), and 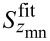
(*f*) is reported in Figure 6c over a 9 Hz wide spectral range. The orange curve in Figure 6c shows the tn-PSD 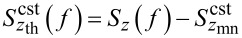, the blue curve shows 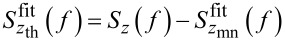, and the green curve shows 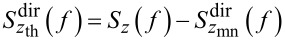.

As mentioned earlier, we use no curve-fitting process for 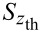
(*f*) and only focus on methods 1 and 3. Method 1 is used with an integration range of ±60 Hz around *f*_1_, where no parasitic peak is visible while ensuring a good accuracy of the stiffness calibration, based on the numerical results. The stiffness thus derived from 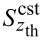
(*f*), 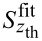
(*f*), and 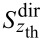
(*f*) gives 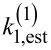 = 1800, 1995, and 318 N/m, respectively. Method 3 applied to 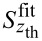
(*f*) gives 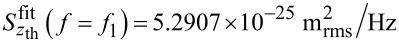, and 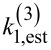 = 2007 N/m. We cannot straightforwardly establish error bars on those measurements at that stage, but this is done hereafter. Therefore methods 1 and 3 applied to 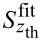
(*f*) are found to be in excellent agreement. As suspected, the estimated stiffness deduced from 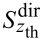
(*f*) is critically underestimated and unreliable. Furthermore, because we have numerically established that the calibration stiffness is more accurate when using a background measurement noise estimated over the entire acquisition bandwidth rather than a constant one, the most realistic stiffness of qPlus 2 is that derived from 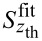
(*f*), namely 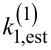 = 1995 N/m. The continuous red curve in Figure 6c is the theoretical tn-PSD of the SHO (Equation 17) calculated with the now established values of *f*_1_, 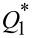, and 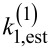. The curve overlays with a good agreement on top of the experimental tn-PSDs over three decades.

Figure 6d is a zoom area on top of the tn-PSD shown in Figure 6c. The frequency range is now 250 mHz. The experimental samples of 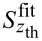
(*f*) are displayed with their error bars of ±10 mHz in frequency, consistent with the frequency resolution, and ±10% in 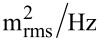, consistent with our amplitude calibration uncertainty. The two thick, light grey curves depict the tn-PSD of the SHO (Equation 17) with 10% deviations of 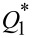 and 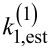. Because the quality factor and the stiffness have opposite contributions in the tn-PSD (cf. Equation 18), the upper curve is obtained with 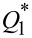 + 10% and 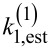 − 10%, and the lower one with 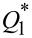 − 10% and 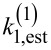 + 10%. It may be seen from the figure that the relevant experimental samples located within the qPlus bandwidth, 
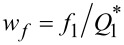
 ≃ 81 mHz, depicted with the shaded rectangle, sit all well within this interval. Therefore, we conclude that the stiffness is estimated with a maximum relative uncertainty of 10%, which is reported in Table 3.

**Table 3:** Estimated stiffness of the two qPlus sensors used in this study. Various methods have been used. When the tip mass correction is applied, one assumes the tip geometry to be the same as that given in Figure 1, which is a strong hypothesis and explains the dispersion between the data (cf. text).

Method	Estimated	Tip mass	qPlus 1 (no tip)	qPlus 2 (with tip)
	stiffness	correction	(N/m)	(N/m)

geometry (Equation 2)	*k*_s_	no	(3322 ± 1270)	not applicable
Lübbe et al. [51] (Equation 4)	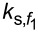	no	(809 ± 162)	not applicable
Cleveland et al. [32] (cf. Supporting Information File 1, Equation S5)	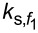	no	(1296 ± 195)	not applicable
Cleveland et al. [32] (cf. Supporting Information File 1, Equation S14)	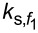	yes	not applicable	(1447 ± 223)
Lozano et al. [49] (cf. Supporting Information File 1, Equation S12)	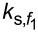	yes	not applicable	(1636 ± 164)

tn-PSD		implicit		
method 1, bkg = 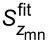 (*f*)	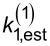		(1289 ± 130)	(1995 ± 200)
method 3, bkg = 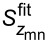 (*f*)	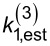		(1544 ± 155) Equation 10	(2007 ± 200) Supporting Information File 1, Equation S13
Equation 10, or Supporting Information File 1, Equation S13, with 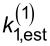	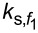		(1252 ± 125)	(3012 ± 301)

The same processing was reproduced with qPlus 1 (*f*_1_ = 29,182.99 Hz and 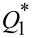 = 172,000) featuring no tip. The estimated stiffness with methods 1 and 3 using 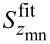
(*f*) as background measurement noise (±60 Hz integration range) gives 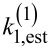 = (1289 ± 130) N/m and 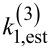 = (1544 ± 155) N/m, respectively. Like with qPlus 2, we note a slightly larger value of 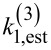 with respect to 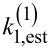. The results are gathered in Table 3 and are discussed in the section below.

## Discussion

In this section, we discuss the advantage of method 1 over method 3, and we compare the thermal noise-based estimated stiffness to values derived from other approaches (cf. Table 3).

Our results indicate that method 3 tends to slightly overestimate the stiffness with respect to method 1. But the latter method forces the experimentalist to point the maximum of the PSD at the resonance, which introduces a somewhat subjective bias in the stiffness estimate. Conversely, method 1 integrates the PSD without subjective input. Thus, any potential statistical bias is smeared out as compared to method 3. This is why method 1 is likely more robust and, hence, preferred to method 3.

It is reminded that qPlus 1 features no tip and that the SEM imaging has been performed with a qPlus sensor that is neither qPlus 1 nor qPlus 2. Thus, we cannot guarantee that the tip dimensions that are used hereafter are consistent with those of the qPlus 2. The discussion is adapted consistently.

For the static stiffness derived from the geometrical parameters of a qPlus sensor (Equation 2, model without tip), we have yet calculated *k*_s_ = (3322 ± 1270) N/m. Lübbe’s approach [51] (Equation 4 with α_1_ = 1.875), leads for qPlus 1 to: 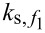 = (809 ± 162) N/m. We also estimate the stiffness from Cleveland’s approach [32] 
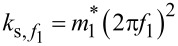
, where 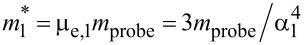 is the effective mass of the fundamental eigenmode (cf. also Supporting Information File 1, Equation S5). With the geometric parameters introduced, the prong mass is estimated to be *m*_probe_ = (160 ± 24) ng (thus, 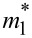 = (39 ± 6) ng). The mass of the metallic electrodes is neglected compared to *m*_probe_. For qPlus 1, Cleveland’s approach [32] leads to 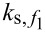 = (1296 ± 195) N/m.

The latter values stand for the qPlus static stiffness and are not strictly comparable to 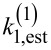 (or 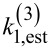), which depict the modal stiffness of the probe’s fundamental eigenmode. Nevertheless, because qPlus 1 features no tip, its static stiffness can be derived from 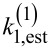 (or 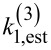) from Equation 10. Then, we get 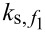 = (1252 ± 125) N/m. Cleveland’s approach [32] and thermal noise measurements are, therefore, in good agreement. However, we notice a strong discrepancy between the former results and the stiffness derived from the geometrical parameters of the qPlus (overestimate) and Lübbe’s approach [51] (underestimate). The reason for that has not been investigated, but both approaches rely on the embedded beam theory. It might be that such a description does not perfectly suit to the type of qPlus sensors investigated here, as discussed in the bibliographic section of the work by Falter and coworkers [65]. Besides, the tip of qPlus 1 has been removed with the goal to have a perfectly free prong, whose behavior might be compared to the geometrical model. But again, we cannot guarantee that there is no glue left at the end of the free prong, which might falsify the comparison to the model.

Regarding qPlus 2, the influence of both tip and glue used to hold it must change its stiffness. This is not accounted for in Equation 2 or Equation 4 and must now be considered. In their article, Cleveland et al. had proposed a simple approach to that problem [32] (cf. also Supporting Information File 1, Equation S14). The stiffness of the loaded prong is 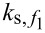 = *m*(2π*f*_1_)^2^, where *m* is not the total mass of the probe including the tip, but given by 
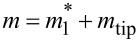
. Doing so, they do not consider the modification of the first eigenmode geometry due to the added mass, which makes the approach approximate. 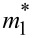 has been established. As for *m*_tip_, based on Figure 1, we assume for the tip an effective cylinder of 50 μm diameter and of *h* = (570 ± 29) μm height (cf. Figure 1b). The mass of the glue is neglected compared to *m*_tip_. We get *m*_tip_ = (22 ± 4) ng. Thus, for qPlus 2, Cleveland’s approach [32] now yields 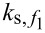 = (1447 ± 223) N/m. The approach by Lozano et al. [49] improves Cleveland’s one. As mentioned, the tip changes the geometry of the probe eigenmodes compared to the unloaded case. This results in a change of the value of the constant α*_n_* of each eigenmode (now becoming 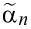, cf. Equation 6). To derive the value of 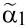 for the first eigenmode, Equation 6 is to be solved with a selected value of μ = *m*_tip_/*m*_probe_. With our estimations, μ ≃ 13.8%. The numerical solution of Equation 6 yields 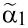 = 1.679. Thus, we derive the stiffness of the equivalent SHO featuring a total mass *m* = *m*_probe_ + *m*_tip_ according to (cf. Supporting Information File 1, Equation S12):


[30]

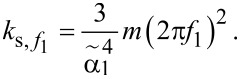



For the qPlus 2, the approach by Lozano et al. [49] yields 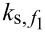 = (1636 ± 164) N/m, closer to 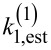 (or 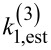), and, thus, more consistent than Cleveland’s one [32]. However, it is again outlined that 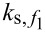 and 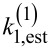 (or 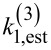) do not depict the same stiffness. The connection between them is not given by Equation 10 anymore, but by:


[31]

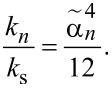



For the qPlus 2, we ultimately get 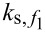 = (3012 ± 301) N/m. The estimated static stiffness is about twice as large as Lozano’s one. However as already mentioned, the tip geometry is not guaranteed; hence, the relevance of the comparison is neither.

At last, a large discrepancy between the estimated modal stiffness values of qPlus 1 and 2 is also noticed, which is assigned to the absence/presence of the tip. Our results highlight both the fundamental role played by the tip in the estimated stiffness and the urge for the stiffness calibration of each qPlus sensor. For us, measuring the tip geometry cannot be performed in situ because the SEM is not connected to the experimental setup. Hence, the interest in thermal noise measurements.

Our final conclusion is that the most relevant value we can rely on for qPlus 2 is its modal stiffness 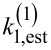 = (1995 ± 200) N/m, determined through thermal noise measurements from method 1. The fact that this value coincides to within 10% of the value of 1800 N/m reported for the early versions of qPlus sensor is purely fortuitous and specific to this qPlus sensor. This value will be different for other qPlus sensors since they will feature different tips.

## Conclusion

This work details a combined numerical and experimental framework for the stiffness calibration (*k*) of a particular type of non-contact atomic force microscopy (nc-AFM) probes, the so-called qPlus sensors, in ultrahigh vacuum (UHV) and at low temperature (9.8 K), based on thermal noise measurements. The qPlus design for which the stiffness calibration is performed is shown in Figure 1. These sensors are based on a quartz tuning fork with one prong fixed and a metallic wire glued at the front end of their free prong forming the tip. The stiffness calibration of such high-*k* sensors, featuring high-quality factors (*Q*) as well, requires to master both the acquisition parameters and the data post-processing. Our approach relies on the statistical analysis of the thermal noise power spectral density (tn-PSD) of the fluctuations of the qPlus sensor’s free prong. The estimated stiffness is derived upon analysis of the tn-PSD by means of the Euler–Bernoulli model and the simple harmonic oscillator equivalence, an otherwise common approach in the literature. Our numerical framework allows us to optimize the experimental acquisition parameters and draw conclusions about the most efficient way to treat the measurement noise that interferes with the tn-PSD. Among several methods introduced for estimating the stiffness from the tn-PSD, it has been shown that the most reliable is to perform the tn-PSD integration over a limited bandwidth around the resonance of the qPlus sensor. The subsequent analysis of the experimental tn-PSD yields a value for the modal stiffness of the qPlus of ≃2000 N/m, with an uncertainty of 10% maximum, whereas the corresponding static stiffness of the sensor featuring no tip, derived from geometric criteria, is 3300 N/m.

Our work highlights the influence of the tip on the estimated stiffness, as well as the need for individual calibration of these probes. Calibrating the stiffness by measuring thermal noise also proves to be more reliable than geometric methods that not only require highly accurate measurements of probe and tip dimensions, but also an ad hoc mechanical model.

Our framework details many technical and practical aspects of the stiffness calibration of qPlus sensors in UHV and at low temperature. From this point of view, it may be adapted to any high-*k*, high-*Q* nc-AFM probe used under similar conditions, such as silicon cantilevers and length extensional resonators, the latter including the KolibriSensor.

Nowadays, a lot of nc-AFM experiments based on qPlus sensors use frequency shift spectroscopy to deduce the forces and potentials above individual atoms or molecules in order to quantitatively evaluate the tip–sample interaction. Most of these publications use a value of 1800 N/m for the stiffness, reported more than 20 years ago for the original qPlus sensor design. However, as we have shown, this can lead to wrong estimates, as the stiffness is used in the frequency shift-to-force conversion.

## Supporting Information

Supporting information features four files. File Supporting Information File 1 reminds the most salient results of the Euler–Bernoulli model and how it sustains the point-mass SHO equivalence. File Supporting Information File 2 reminds fundamental elements of signal processing applied to discrete time signals, among which the Power Spectral Density. File Supporting Information File 3 reminds the expression of the thermal noise PSD of a SHO in thermal equilibrium within a thermostat. The PSD of the stochastic thermal force giving rise to the fluctuations of the SHO is derived as well, which is used in the numerical simulations. File Supporting Information File 4 illustrates the influence of a digital antialiasing filter on the measured tn-PSD.

File 1 The Euler–Bernoulli model and the point-mass SHO equivalence

File 2 Discrete Fourier transform and power spectral density of a discrete time signal

File 3 Power Spectral densities of a SHO and of the thermal force – Probability density function of the thermal force

File 4 Influence of a digital antialiasing filter on the measured tn-PSD

## Data Availability

The data that supports the findings of this study is available from the corresponding author upon reasonable request.
