# Supplementary material for: Stiffness calibration of qPlus sensors at low temperature through thermal noise measurements
Source: Beilstein J Nanotechnol. 2024 May 23;15:580–602. doi: 10.3762/bjnano.15.50 (PMC11181211; doi:10.3762/bjnano.15.50)
Supplement: File 1 — The Euler–Bernoulli model and the point-mass SHO equivalence [file Beilstein_J_Nanotechnol-15-580-s001.pdf]

BEILSTEIN JOURNAL OF NANOTECHNOLOGY

Supporting Information

for

Stiffness calibration of qPlus sensors at low temperature through thermal noise measurements

Laurent Nony, Sylvain Clair, Daniel Uehli, Aitziber Herrero, Jean-Marc Themlin, Andrea Campos, Franck Para, Alessandro Pioda and Christian Loppacher

Beilstein J. Nanotechnol. **2024,** *15,* 580–602. doi:10.3762/bjnano.15.50

The Euler–Bernoulli model and the point-mass SHO equivalence

License and Terms: This is a supporting information file under the terms of the Creative Commons Attribution License (https://creativecommons.org/licenses/by/4.0). Please note that the reuse, redistribution and reproduction in particular requires that the author(s) and source are credited and that individual graphics may be subject to special legal provisions.
The license is subject to the *Beilstein Journal of Nanotechnology* terms and conditions: (https://www.beilstein-journals.org/bjnano/terms)

The Euler-Bernoulli's model and the point-mass SHO equivalence

This SI file details some of the elements discussed in the literature about the equivalence between the Euler-Bernoulli's model (continuous beam theory) and the point-mass simple harmonic oscillator (SHO) model for each eigenmode of a probe. As discussed by Melcher *et al.* [1], due to different choices of eigenmode scaling and normalization, some mass and stiffness values reported in the literature may seem inconsistent. This document relies on three references to focus the discussion [1-3].

The Euler-Bernoulli's model accounts for the deflection of any flexural eigenmode of the probe anywhere along its longitudinal axis (*e.g.* x, *cf.* fig.1 in the main text), *i.e.* in particular at its free end ($x = L$). The spatial and temporal evolution of the deflection at any position x may be described by that of an equivalent SHO of resonance frequency f_n, stiffness k_n, mass m_n, and Q-factor Q_n. The Q-factor reflects the damping of the probe in the medium within which it oscillates. We specifically focus here at the equivalent SHO at the prong free end $x = L$. This model is of central importance when dealing with thermal noise measurements because the resulting deflection of the probe free end is modeled as the superposition of the deflections of a virtually infinite number of probe eigenmodes (modal decomposition).

Melcher *et al.* postulate that a true equivalent point-mass model must necessarily possess the same elastic strain, kinetic, and tip-sample interaction energies as the continuous probe for a given eigenmode. This must lead to unique equivalent mass and stiffness values for the point-mass model and guarantees that the point-mass model and the continuous probe possess identical tip displacements and tip-sample interaction forces.

The calculations of the solutions functions of the Euler-Bernoulli's problem are detailed in refs. [2,4]. As mentioned in the main text, we assume a rectangular shaped cross-section of the probe featuring an homogeneous mass distribution, the value of which is given by its density ρ, along with its geometric dimensions (l, t, w): $m_{\text{probe}} = \rho l t w$. Melcher *et al.* then established the resonance frequency of the equivalent point-mass SHO model of the nth probe eigenmode according to:

$$f_n = \frac{1}{2\pi}\sqrt{\frac{\alpha_n^4 k_s}{3m_{\text{probe}}}} = \frac{1}{2\pi}\sqrt{\frac{k_n}{m_n}}, \tag{S1}$$

where:

$$m_n = \frac{m_{\text{probe}}}{4} \tag{S2}$$

and:

$$\frac{k_n}{k_s} = \frac{\alpha_n^4}{12} \tag{S3}$$

It is also interesting to interpret equ.S1 as follows:

$$f_n = \frac{1}{2\pi}\sqrt{\frac{\alpha_n^4 k_s}{3m_{\text{probe}}}} = \frac{1}{2\pi}\sqrt{\frac{k_s}{m_n^*}} \tag{S4}$$

Then, noting $k_s \rightarrow k_{s,f_n}$:

$$k_{s,f_n} = m_n^*(2\pi f_n)^2 = \frac{3}{\alpha_n^4} m_{\text{probe}}(2\pi f_n)^2 \tag{S5}$$

The static stiffness of the probe k_{s,f_n} may be estimated out of the resonance frequency f_n of the nth eigenmode depicting an equivalent SHO of effective mass $m_n^* = 3m_{\text{probe}}/\alpha_n^4$. This is Cleveland's [5], or Lübbe's [6] framework (equ.5 in the reference). For the fundamental eigenmode $n = 1$ of an unloaded probe, $\alpha_1 = 1.875$, and $m_1^* = 0.2427m_{\text{probe}}$, hence the effective normalized mass $\mu_{e,1} = 0.2427$ mentioned in the main text.

These elements allow us to derive the relationship between f_n and the thickness of the probe, which ultimately leads to the static stiffness derived by Cleveland *et al.* [5], and later on by Lübbe *et al.* [6], namely:

$$f_n = \frac{1}{2\pi}\sqrt{\frac{\alpha_n^4 k_s}{3m_{\text{probe}}}} \overbrace{=}^{\textit{cf.}\ \text{equ.2 main text, and } m_{\text{probe}}=\rho l t w} \frac{\alpha_n^2 t}{2\pi l^2}\sqrt{\frac{E}{12\rho}} \tag{S6}$$

By expressing t as a function of f_n and introducing that dependence into equ.2 of the main text, we get:

$$k_{s,f_n} = \frac{2w(\pi l f_n)^3}{\alpha_n^6}\sqrt{\frac{12^3\rho^3}{E}}, \tag{S7}$$

which is the expression by Lübbe *et al.* (*cf.* equ.4 of the main text). For the fundamental eigenmode, $n = 1$, we get:

$$k_{s,f_1} = \frac{2w(\pi l f_1)^3}{\alpha_1^6}\sqrt{\frac{12^3\rho^3}{E}} \tag{S8}$$

Because $\sqrt{12^3}/\alpha_1^6 \simeq 1$, the above expression supports Cleveland's equation (*cf.* equ.3 of the main text), while improving it.

Equation S1 permits to connect the resonance frequency of the nth eigenmode, f_n, and the fundamental one ($n = 1$), f_1, which is also found *e.g.* in ref.[4]:

$$\frac{f_n}{f_1} = \left(\frac{\alpha_n}{\alpha_1}\right)^2 \tag{S9}$$

In particular, $f_2/f_1 \simeq 6.27$. For a qPlus sensor with $f_1 \simeq 25$ kHz, then $f_2 \simeq 156.750$ kHz. Because

the probe undergoes no viscous damping in UHV, the quality factor Q_n of each eigenmode only stems from the intrinsic damping of the probe which is related to the material of which it is made. It is also reminded that in the SHO formalism, the damping is responsible for the enlargement of the resonance curve, defining the *SHO bandwidth* w_f, *i.e.* the width of the resonance curve estimated at $A_1/\sqrt{2}$, if A_1 is the resonance amplitude. Q_1 is then defined according to $Q_1 = f_1/w_f$. For qPlus sensors in LT UHV, Q_1 values of up to 2.10^5 can be achieved. Thus, $w_f = f_1/Q_1 \simeq 125$ mHz only. Thus, it is often stated, even in the case of non-UHV measurements, that each eigenmode features a Q-factor Q_n that is large enough to avoid any spectral overlap in the frequency response of two subsequent eigenmodes. The set of SHO's describing the mechanical behavior of the qPlus are then assumed to be independent.

Equation S1 immediately gives the connection between the modal stiffness of the nth mode and the fundamental one k_1:

$$\frac{k_n}{k_1} = \left(\frac{f_n}{f_1}\right)^2 = \left(\frac{\alpha_n}{\alpha_1}\right)^4 \tag{S10}$$

In particular, $k_2/k_1 \simeq 39.3$. For a qPlus sensor with $k_1 \simeq 1800$ N/m, then $k_2 \simeq 70.8$ kN/m.

The above framework does not consider the influence of the tip mass added at the prong free end. But the model can be extended to that case. The structure of the solutions functions of the Euler-Bernoulli's model remain unchanged [3], it is only the equation for α_n (now noted $\widetilde{\alpha}_n$ to discriminate it from the unloaded case) that is changed to account for the tip mass through the variable $\mu = m_{\text{tip}}/m_{\text{probe}}$ (*cf.* equ.6 of the main text). The nth equivalent SHO now features an actual total mass $m = m_{\text{tip}} + m_{\text{probe}}$ and, following the same argumentation, the corresponding resonance frequency is given by:

$$f_n = \frac{1}{2\pi}\sqrt{\frac{\widetilde{\alpha}_n^4 k_s}{3m}} \tag{S11}$$

Thus:

$$k_{s,f_n} = \frac{3}{\widetilde{\alpha}_n^4}(m_{\text{tip}} + m_{\text{probe}})(2\pi f_n)^2 \tag{S12}$$

Note that the framework also modifies the relation between k_n and k_s (*cf.* equ.S3 in this document, or equ.10 of the main text), now written as:

$$\frac{k_n}{k_s} = \frac{\widetilde{\alpha}_n^4}{12} \tag{S13}$$

At last, note also that Cleveland's approach in the case of the loaded probe [5] is approximative as it gives (equ.4 in the reference):

$$k_{s,f_n} = \left(\frac{3}{\alpha_n^4} m_{\text{probe}} + m_{\text{tip}}\right)(2\pi f_n)^2 \tag{S14}$$

References

1. Melcher, J.; Hu, S.; Raman, A. *Appl. Phys. Lett.* **2007**, *91*, 053101. doi:10.1063/1.2767173.

2. Butt, H.-J.; Jaschke, M. *Nanotechnology* **1995**, *6*, 1–7. doi:10.1088/0957-4484/6/1/001.

3. Lozano, J.; Kiracofe, D.; Melcher, J.; Garcia, R.; Raman, A. *Nanotechnology* **2010**, *21*, 465502. doi:10.1088/0957-4484/21/46/465502.

4. Paolino, P.; Tiribilli, B.; Bellon, L. *J. Appl. Phys.* **2009**, *106*, 094313. doi:10.1063/1.3245394.

5. Cleveland, J. P.; Manne, S.; Bocek, D.; Hansma, P. K. *Rev. Sci. Instrum.* **1993**, *64*, 403–405. doi:10.1063/1.1144209.

6. Lübbe, J.; Doering, L.; Reichling, M. *Meas. Sci. Technol.* **2012**, *23*, 045401. doi:10.1088/0957-0233/23/4/045401.
