# Supplementary material for: Stiffness calibration of qPlus sensors at low temperature through thermal noise measurements
Source: Beilstein J Nanotechnol. 2024 May 23;15:580–602. doi: 10.3762/bjnano.15.50 (PMC11181211; doi:10.3762/bjnano.15.50)
Supplement: File 2 — Discrete Fourier transform and power spectral density of a discrete time signal [file Beilstein_J_Nanotechnol-15-580-s002.pdf]

BEILSTEIN JOURNAL OF NANOTECHNOLOGY

Supporting Information

for

Stiffness calibration of qPlus sensors at low temperature through thermal noise measurements

Laurent Nony, Sylvain Clair, Daniel Uehli, Aitziber Herrero, Jean-Marc Themlin, Andrea Campos, Franck Para, Alessandro Pioda and Christian Loppacher

Beilstein J. Nanotechnol. **2024,** *15,* 580–602. doi:10.3762/bjnano.15.50

Discrete Fourier transform and power spectral density of a discrete time signal

License and Terms: This is a supporting information file under the terms of the Creative Commons Attribution License (https://creativecommons.org/licenses/by/4.0). Please note that the reuse, redistribution and reproduction in particular requires that the author(s) and source are credited and that individual graphics may be subject to special legal provisions.
The license is subject to the *Beilstein Journal of Nanotechnology* terms and conditions: (https://www.beilstein-journals.org/bjnano/terms)

Discrete Fourier Transform & Power Spectral Density of a discrete time signal

This SI file details the definitions of the variables and the concepts used in this work. These elements cover fundamental aspects of signal processing applied to discrete time signals. They are inspired by the book by E.O. Brigham [1]. Particular attention is paid to the physical units of the variables.

Discrete Fourier Transform

Let's assume a generic, square summable, continuous time signal $x(t)$ forming a Fourier pair:

$$x(t) \triangleq \int_{-\infty}^{+\infty} \hat{x}(f)e^{+j2\pi ft}df \rightleftharpoons \hat{x}(f) \triangleq \int_{-\infty}^{+\infty} x(t)e^{-j2\pi ft}dt \tag{S1}$$

The frequency spectrum of the Fourier transform is continuous and two-sided ($f \in \mathbb{R}$). The physical units of $x(t)$ and $\hat{x}(f)$ are inequivalent. If $x(t)$ is recorded in m (or V), then $\hat{x}(f)$ is expressed in m.s (V.s), or m.Hz^{-1} (V.Hz^{-1}).

Practically, $x(t)$ is always sampled over a finite windowing duration T_w, which is usually assumed to be causal as well, hence $t \in [0; T_w[$.

The discrete time version of that windowed signal is obtained upon sampling with a sampling period T_s (sampling frequency $f_s = 1/T_s$) within the time interval $[0; T_w[$. This results in N samples for the signal and for its time support: $t \longrightarrow t_n = (n-1)T_s$, and $x(t) \longrightarrow x_n$, $\forall n \in [1; N], n \in \mathbb{N}^*$. T_w, T_s and N are now connected according to:

$$T_w = NT_s \tag{S2}$$

The sampling process along with the time truncation of the signal sets the framework of the Discrete Fourier Transform (DFT) [1,2], whose numerical implementation in most of scientific softwares is

performed out of the function `fft` (*e.g.* Matlab). We hereafter clarify some key points for a correct use of this tool.

The Fourier pair of the sampled, windowed, signal x_n (hereafter referred to as the *sampled signal* only) is now:

$$x_n \rightleftharpoons \hat{x}_n, \tag{S3}$$

where its DFT $\hat{x}_n$ is defined as:

$$\hat{x}_n \triangleq \frac{1}{N}\mathrm{fft}(x_n) \tag{S4}$$

$\hat{x}_n$ features N samples that spread in a **two-sided, bounded, frequency domain** satisfying the Shannon-Nyquist criterion $f \in [-f_s/2; f_s/2[$, whose discrete expression is:

$$f \longrightarrow f_n = -f_s/2 + (n-1)\delta f, \forall n \in [1; N] \tag{S5}$$

δf is the spectral resolution of the spectrum:

$$\delta f = \frac{f_s}{N} = \frac{1}{NT_s} = \frac{1}{T_w} \tag{S6}$$

The time (frequency) windowing within $[0; T_w[$ ($[-f_s/2; +f_s/2[$), along with the time (frequency) sampling (N samples) makes x_n T_w-periodic and $\hat{x}_n$ f_s-periodic. The samples of the DFT $\hat{x}_n$ may therefore be interpreted as the **Fourier series coefficients of the signal** x_n, and the physical units of both sampled signals are now the same.

To ensure the good consistency between the N samples of the frequency support f_n and the DFT samples, it is important to figure out how these are returned by the function `fft`. In most of its implementations, the function returns N samples whose $N/2$ first stand for the Fourier series coefficients being part of the $[0; f_s/2[$ frequency interval. The $N/2$ subsequent samples stand for the Fourier series coefficients being part of the $[-f_s/2; 0[$ frequency interval. Thus, for a consistent two-sided representation of the DFT with the frequency support f_n, it is actually mandatory to shift the upper half part of the DFT samples to the lower half part of the array, which is usually performed by the function `fftshift` (*e.g.* Matlab).

The two-sided representation of the DFT forms a strict, self-consistent, mathematical background, but its one-sided representation may be equivalently used. Then the **one-sided frequency spectrum** of the DFT is used instead by selecting the $N/2$ first samples of $\hat{x}_n$, as returned by `fft`. To account for the components in the negative part of the frequency axis, owing the Hermitian property of the DFT: $\hat{x}(-f) = \hat{x}^*(f)$, the DFT samples are multiplied by a factor of 2, except the one at null frequency (mean value, or DC component, of the signal). Algorithmically, this summarizes to:

$$\hat{x}_{n_L} = \begin{cases} \hat{x}_1 \\ 2\hat{x}_n, \forall n \in [2; N/2] \end{cases} \tag{S7}$$

The one-sided frequency support is now defined as:

$$f_{n_L} = (n-1)\delta f, \forall n \in [1; N/2] \tag{S8}$$

Power Spectral Density of a discrete time signal

The mean power of a sampled signal x_n is defined as the time-averaged of its squared value:

$$P_x \triangleq \langle x_n^2 \rangle_{T_w} = \frac{1}{T_w} \sum_{n=1}^{N} x_n^2 T_s = \frac{1}{N} \sum_{n=1}^{N} x_n^2 \tag{S9}$$

The definition of the power specifies the rms value, x_n^{rms}, of the signal as well:

$$x_n^{\text{rms}} \triangleq \sqrt{P_x} = \sqrt{\langle x_n^2 \rangle_{T_w}} \tag{S10}$$

The analysis of stochastic signals is usually easier when performed on their spectrum, rather than on their time trace. It is then convenient to define the mean power of the sampled signal x_n out of its elemental spectral quantity, the Power Spectral Density (PSD, notation: $S_x(f_n)$):

$$P_x \triangleq \sum_{n=1}^{N} S_x(f_n) \delta f, \tag{S11}$$

where it is reminded that the summation over $n \in [1; N]$ features a two-sided PSD spectrum, with $f_n \in [-f_s/2; f_s/2[$, given by equ.S5. The combination between the two former equations and the Parseval theorem leads to the expression of the discrete *regular PSD* of the sampled signal x_n:

$$S_x(f_n) = |\hat{x}_n|^2 T_w = \frac{|\hat{x}_n|^2}{\delta f} \tag{S12}$$

Assuming a signal $x(t)$ whose units are m (or V), the PSD units are m^2.s (V^2.s), that is $m^2.Hz^{-1}$ ($V^2.Hz^{-1}$), as most commonly used.

However, the observables that are measured from stochastic signals usually relate to rms values (*e.g.* m_{rms} or V_{rms}). It is therefore preferable to express the PSD out of the *rms values of the Fourier series coefficients*, rather than out of their peak values, as implicitly stated by equ.S4:

$$\hat{x}_n^{\text{rms}} = \frac{\hat{x}_n}{\sqrt{2}} \tag{S13}$$

Then, the corresponding PSD, so-called *rms PSD*, becomes:

$$S_x^{\text{rms}}(f_n) \triangleq |\hat{x}_n^{\text{rms}}|^2 T_w = \frac{S_x(f_n)}{2} = \frac{|\hat{x}_n|^2 T_w}{2}, \tag{S14}$$

whose units are $\text{m}_{\text{rms}}^2.\text{Hz}^{-1}$ (or $\text{V}_{\text{rms}}^2.\text{Hz}^{-1}$).

The PSD is a powerful tool to see the spectral density of noise in a signal. It is commonly used in many applications and is mostly represented with a one-sided spectrum. In this work, we have systematically used that type of representation, that relies on the one-sided representation of the DFT $\hat{x}_{n_{\text{L}}}$ of the sampled signal x_n (equ.S7) against its one-sided frequency support $f_{n_{\text{L}}}$ (equ.S8):

$$S_x^{\text{rms}}(f_{n_{\text{L}}}) \triangleq |\hat{x}_{n_{\text{L}}}^{\text{rms}}|^2 T_w = \frac{|\hat{x}_{n_{\text{L}}}|^2 T_w}{2} \forall n \in [2; N/2] \tag{S15}$$

It should be noted that, owing to equ.S7, equ.S15 also leads to: $S_x^{\text{rms}}(f_{n_{\text{L}}}) = |\hat{x}_{n_{\text{L}}}|^2 T_w/2 = 2|\hat{x}_n|^2 T_w = 2S_x(f_n)$. The one-sided rms PSD of a signal may be derived as twice its two-sided regular PSD. Lastly, if the mean value of the sampled signal is 0, which is the case in the present work, equ.S15 stands for all $n \in [1; N/2]$.

References

1. Brigham, E. O. *The Fast Fourier Transform and Its Applications*; Prentice Hall, 1988.

2. https://hal.science/hal-04075823
