# Supplementary material for: Stiffness calibration of qPlus sensors at low temperature through thermal noise measurements
Source: Beilstein J Nanotechnol. 2024 May 23;15:580–602. doi: 10.3762/bjnano.15.50 (PMC11181211; doi:10.3762/bjnano.15.50)
Supplement: File 3 — Power Spectral densities of a SHO and of the thermal force – Probability density function of the thermal force [file Beilstein_J_Nanotechnol-15-580-s003.pdf]

BEILSTEIN JOURNAL OF NANOTECHNOLOGY

Supporting Information

for

Stiffness calibration of qPlus sensors at low temperature through thermal noise measurements

Laurent Nony, Sylvain Clair, Daniel Uehli, Aitziber Herrero, Jean-Marc Themlin, Andrea Campos, Franck Para, Alessandro Pioda and Christian Loppacher

Beilstein J. Nanotechnol. **2024,** *15,* 580–602. doi:10.3762/bjnano.15.50

Power Spectral densities of a SHO and of the thermal force – Probability density function of the thermal force

License and Terms: This is a supporting information file under the terms of the Creative Commons Attribution License (https://creativecommons.org/licenses/by/4.0). Please note that the reuse, redistribution and reproduction in particular requires that the author(s) and source are credited and that individual graphics may be subject to special legal provisions.
The license is subject to the *Beilstein Journal of Nanotechnology* terms and conditions: (https://www.beilstein-journals.org/bjnano/terms)

Power Spectral Densities of a SHO and of the thermal force - Probability density function of the thermal force

This SI file relies on the elements developed in the SI file 2 to derive the expression of the thermal noise PSD of a SHO in thermal equilibrium within a thermostat carrying a thermal energy $k_B T$. The PSD of the stochastic, thermal force giving rise to the fluctuations of the SHO is derived as well. This allows us to derive the standard deviation (rms value) of the thermal force, along with its probability density, used in the numerical simulations. The elements below are a digest of several sources [1,2].

Power Spectral Densities of a SHO and of the thermal force

In this work, the prong of the QTF behaves as a SHO (resonance frequency f_1, Q-factor Q_1, stiffness k_1) subject to thermal fluctuations. Assuming $F_{\text{th}}(t)$ to be the instantaneous value of the thermal force acting on the SHO, the instantaneous position of the prong in time, $z(t)$, obeys the classical second order differential equation:

$$m_1 \ddot{z}(t) + m_1 \frac{2\pi f_1}{Q_1} \dot{z}(t) + k_1 z(t) = F_{\text{th}}(t) \tag{S1}$$

At that stage, $z(t)$ and $F_{\text{th}}(t)$ stand for continuous time signals, whose corresponding Fourier pairs, under our Fourier transform convention (*cf.* SI file 2, equ.1), are: $z(t) \rightleftharpoons \hat{Z}(f)$ and $F_{\text{th}}(t) \rightleftharpoons \hat{F}_{\text{th}}(f)$. The Fourier transform of the above equation then gives:

$$\hat{Z}(f)\hat{\chi}_{\text{SHO}}(f) = \hat{F}_{\text{th}}(f), \tag{S2}$$

where $\hat{\chi}_{\text{SHO}}(f)$ is the Fourier transform of the susceptibility, or linear response function, of the SHO:

$$\hat{\chi}_{\text{SHO}}(f) = k_1 \left[1 - \left(\frac{f}{f_1} \right)^2 + j \frac{f}{Q_1 f_1} \right] \tag{S3}$$

Equation S2 illustrates that $\hat{F}_{\text{th}}(f)$ is characterized, to first order, by the Fourier transform of the linear response function of the SHO.

This sets the framework of the Fluctuation–Dissipation Theorem (FDT) [3,4] that relates the strength of the thermal fluctuations the SHO undergoes to its dissipation, here characterized by its quality factor Q_1, and whose frequency response is quantified by $\hat{\chi}_{\text{SHO}}(f)$. The FDT relates the two-sided PSD of $z(t)$ and $F_{\text{th}}(t)$, namely $S_z(f)$ and $S_{F_{\text{th}}}(f)$ respectively, to the imaginary part of $\hat{\chi}_{\text{SHO}}(f)$. On the one hand:

$$S_z(f) = -\frac{k_B T}{\pi f} \text{Im} \left\{ \frac{1}{\hat{\chi}_{\text{SHO}}(f)} \right\}, \tag{S4}$$

and on the other hand:

$$S_{F_{\text{th}}}(f) = +\frac{k_B T}{\pi f} \text{Im} \left\{ \hat{\chi}_{\text{SHO}}(f) \right\} \tag{S5}$$

Equation S4 leads to:

$$S_z(f) = \frac{k_B T}{\pi k_1 Q_1 f_1} \frac{1}{\left[1 - \left(\frac{f}{f_1} \right)^2 \right]^2 + \left(\frac{f}{Q_1 f_1} \right)^2}, \tag{S6}$$

whereas equ.S5 leads to:

$$S_{F_{\text{th}}}(f) = \frac{k_B T k_1}{\pi Q_1 f_1} \tag{S7}$$

The former equations stand for continuous time signals. If both signals $z(t)$ and $F_{\text{th}}(t)$ are now T_s-sampled over a duration T_w, following the description detailed in the SI file 2, then their corresponding **one-sided rms PSD** are written:

$$S_z^{\text{rms}}(f_{n_\text{L}}) = \frac{2k_B T}{\pi k_1 Q_1 f_1} \frac{1}{\left[1 - \left(\frac{f_{n_\text{L}}}{f_1}\right)^2\right]^2 + \left(\frac{f_{n_\text{L}}}{Q_1 f_1}\right)^2}, \tag{S8}$$

and:

$$S_{F_{\text{th}}}^{\text{rms}}(f_{n_\text{L}}) = \frac{2k_B T k_1}{\pi Q_1 f_1} \tag{S9}$$

It is reminded that $S_z^{\text{rms}}(f_{n_\text{L}})$ and $S_{F_{\text{th}}}^{\text{rms}}(f_{n_\text{L}})$ are sampled signals consisting of $N/2$ samples and that $f_{n_\text{L}} \in [0; f_s/2[$, as detailed in the SI file 2, equ.8.

Probability density function of the thermal force

Equation S9 allows us to specify the probability density function of the thermal force that is used in the numerical simulations. Indeed, the rms value of the thermal force fluctuations $F_{\text{th}}^{\text{rms}}$ in a time interval $t \in [0; T_w[$ representing a frequency interval $[0; f_s/2[$, is given by:

$$
\begin{aligned}
F_{\text{th}}^{\text{rms}} &\triangleq \sqrt{P_{F_{\text{th}}}} = \sqrt{\langle F_{\text{th}}^2 \rangle_{T_w}} \\
&= \sqrt{\textstyle\sum_{n=1}^{N/2} S_{F_{\text{th}}}^{\text{rms}}(f_{n_\text{L}})\delta f} \qquad \text{(S10)} \\
&= \sqrt{\tfrac{2k_BTk_1}{\pi Q_1 f_1}\tfrac{N\delta f}{2}} = \sqrt{\tfrac{2k_BTk_1}{\pi Q_1 f_1}\tfrac{f_s}{2}}
\end{aligned}
$$

The latter equation may sometimes be found in the literature written as [5]:

$$
F_{\text{th}}^{\text{rms}} = \sqrt{\frac{2k_BTk_1}{\pi Q_1 f_1}B}, \qquad \text{(S11)}
$$

with B, 'the measurement bandwidth'. This statement is quite elusive as B is never clearly correlated to the sampling frequency of the problem. Here, we will keep in mind that $B = f_s/2$ **matches the Nyquist frequency of the problem**.

The stochastic, thermal, force used in the numerical simulations will therefore feature a probability distribution following a normal law (implemented with the function `randn` in Matlab) with a standard deviation $\sigma = F_{\text{th}}^{\text{rms}}$, and centered around 0 (no mean force), hence a probability density function (Pdf):

$$
\text{Pdf}(F_{\text{th}}) = \frac{1}{\sigma\sqrt{2\pi}} e^{-\frac{1}{2}\frac{F_{\text{th}}^2}{\sigma^2}} \qquad \text{(S12)}
$$

References

1. Cook, S. M.; Schaeffer, T. E.; Chynoweth, K. M.; Wigton, M.; Simmonds, R. W.; Lang, K. M. *Nanotechnology* **2006**, *17*, 2135–2145. doi:10.1088/0957-4484/17/9/010.

2. Bellon, L. Exploring nano-mechanics through thermal fluctuations. Habilitation á Diriger des Recherches, Ecole Normale Supérieure de Lyon, 2010.

3. Kubo, R. *Reports on Progress in Physics* **1966**, *29* (1), 255. doi:10.1088/0034-4885/29/1/306.

4. Coleman, P. Fluctuation-dissipation theorem and linear response theory. In *Introduction to Many-Body Physics*; Cambridge University Press, 2015; Chapter 9, pp 292–331.

5. Chps.2 and 20 in ref.[1] of the main text, Chp.15 in ref.[2] of the main text.
