# Supplementary material for: Stiffness calibration of qPlus sensors at low temperature through thermal noise measurements
Source: Beilstein J Nanotechnol. 2024 May 23;15:580–602. doi: 10.3762/bjnano.15.50 (PMC11181211; doi:10.3762/bjnano.15.50)
Supplement: File 4 — Influence of a digital antialiasing filter on the measured tn-PSD [file Beilstein_J_Nanotechnol-15-580-s004.pdf]

BEILSTEIN JOURNAL OF NANOTECHNOLOGY

Supporting Information

for

Stiffness calibration of qPlus sensors at low temperature through thermal noise measurements

Laurent Nony, Sylvain Clair, Daniel Uehli, Aitziber Herrero, Jean-Marc Themlin, Andrea Campos, Franck Para, Alessandro Pioda and Christian Loppacher

Beilstein J. Nanotechnol. **2024,** *15,* 580–602. doi:10.3762/bjnano.15.50

Influence of a digital antialiasing filter on the measured tn-PSD

License and Terms: This is a supporting information file under the terms of the Creative Commons Attribution License (https://creativecommons.org/licenses/by/4.0). Please note that the reuse, redistribution and reproduction in particular requires that the author(s) and source are credited and that individual graphics may be subject to special legal provisions.
The license is subject to the *Beilstein Journal of Nanotechnology* terms and conditions: (https://www.beilstein-journals.org/bjnano/terms)

Influence of a digital antialiasing filter on the measured tn-PSD

In addition to the resonance peak at f_1, the black curve in fig.S1a below (similar to fig.3a in the main text) exhibits two additional, unexpected, peaks looking like resonance peaks, at $f_a \simeq 433$ Hz and $f_b \simeq 31.7$ kHz. However, their magnitudes is much smaller than that of the f_1-peak, by several decades. These peaks are the aliases of the eigenmodes 2 and 3 of the qPlus due to spectral aliasing. Indeed, because f_2 and f_3 do not fulfill the Shannon-Nyquist sampling theorem ($f_2 = 156\,680$ Hz and $f_3 = 438\,650$ Hz, *cf.* table 1 in the main text, thus $f_{2,3} > f_s/2 = B_s = 78.125$ kHz), the peaks fold back within the $[0; B_s]$ interval by aliasing with an apparent frequency: $f_{\text{app}} = f_n - qB_s$ ($n = 2, 3$), if q, the integer quotient of the division of f_n by B_s is even, or: $f_{\text{app}} = B_s(1 + q) - f_n$, if q is odd. The f_2-spectral component is then detected with an apparent frequency $f_{\text{app}} = f_a \simeq 433.6$ Hz and the f_3-spectral component with an apparent frequency $f_{\text{app}} = f_b \simeq 30.1$ kHz. The discrepancy between the numerically-observed apparent frequency f_b and the expected one is attributed to the lack of numerical resolution due to $f_{s,\text{max}}^{\text{num}} = 3.125$ MHz.

After conversion by the charge amplifier, the thermal fluctuations of the qPlus are filtered by the OC4 that features an analog antialiasing filter with a 5 MHz cut-off, which will not prevent the spectral aliasing of the digital signals from the band $[B_s; 5\text{ MHz}]$ to occur into the band $[0; B_s]$. But, apart from that filter, in the digital domain, it is reminded that the acquisition of the thermal noise is performed without additional digital antialiasing filter.

To assess the potential influence of the spectral aliasing on the estimated stiffness, we simulate the influence an additional *digital* antialiasing filter, implemented as a 40th-order Finite Impulse Response (FIR) Low-Pass (LP) filter, may have on the stiffness calibration. The filter has been arbitrarily designed to have a -3 dB attenuation at 51 900 Hz (close to the Nyquist criterion $2 \times f_1$) and -7.3 dB at B_s. The data processing is similar as before, except that now, the $f_{s,\text{max}}$-sampled data are filtered prior to being downsampled. The resulting tn-PSD of the filtered data is displayed as the blue curve in fig.S1a (*cf.* also fig.S1b, spectral range 30 Hz). One has also reported in red the corresponding PSD for the SHO with the parameters $(f_1; Q_1; k_1)$=(25 kHz;10^5;1800 N/m). The influence of the spectral antialiasing and the role of the LP-filter are well-visible in the low-frequency

part of the spectrum (*e.g.* [0..10] kHz range). Indeed, a regular LP-filter applied on well-sampled data, without occurrence of spectral aliasing, should have no influence on them in that range. The fact that in this part of the spectrum the PSD of the filtered data (blue curve) is located below that of the unfiltered data (black curve) testifies that the difference actually stems from high-frequency, LP-filtered components. The ground noise now matches that of the theoretical tn-PSD (continuous red curve).

The same analysis performed with the filtered data leads to the following values of the estimated stiffness (*cf.* table S1 below): $k_{1,\text{est}}^{(1)} = k_{1,\text{est}}^{(2)} = 2058$ N/m, and $S_{z_{th}}(f = f_1) = 1.650\ 10^{-25}\ \text{m}_{\text{rms}}^2/\text{Hz}$, *i.e.* $k_{1,\text{est}}^{(3)} = 2078$ N/m. These values are $\simeq 11$ % larger than the nominal k_1 value.

To compare further filtered and unfiltered (raw) data, the following residuals are built: $\Delta_{\text{raw, filt.}} = \text{PSD}_{\text{raw,filt.}}(f) - \text{PSD}_{\text{SHO}}(f)$. They are reported in fig.S1c over the same spectral range as in fig.S1b. In the vicinity of the resonance, the raw data have a residual smaller in absolute value than the filtered ones, and the residual of the filtered data is negative, indicating that the PSD is underestimated, which in turn yields to an overestimate value of the stiffness.

The discrepancy between the filtered and the unfiltered data stems from the design of the antialiasing filter. If an antialiasing filter is used for the acquisition of the thermal fluctuations, its cut-off frequency must be judiciously adjusted not to affect the estimated stiffness (likely larger than $2 \times f_1$, as chosen here), unless otherwise its influence might be deleterious. On the other hand, if no filter is used, depending on the Q-factor of the aliased components, the estimated stiffness might be affected too. In this case, the noise floor will never match the theoretical noise floor of the SHO either. These effects are expected to be all the more salient with weak Q-factors. In the present case, we get a good estimated stiffness with the unfiltered data because the large Q-factors force the magnitudes of the folded components to be weak-enough not to influence much the resulting PSD.

Table S1: Calibrated stiffness using methods 1, 2 or 3 for unfiltered- (raw) and filtered- (antialiasing, *cf.* text) data. The number of averaged time traces is $M_{\text{num}} = 64$ in each case. Depending on the design of the antialiasing filter, the calibration accuracy may be degraded.

Method (n)	Nominal k_1 (N/m)	$k_{1,\text{est}}^{(n)}$, raw data (N/m) (rel. err.)	$k_{1,\text{est}}^{(n)}$, filt. data (N/m) (rel. err.)
**1**	1800	1750 (-2.8 %)	2058 (+11.4 %)
**2**	1800	1765 (-1.9 %)	2060 (+11.4 %)
**3**	1800	1813 (+0.7 %)	2087 (+11.6 %)

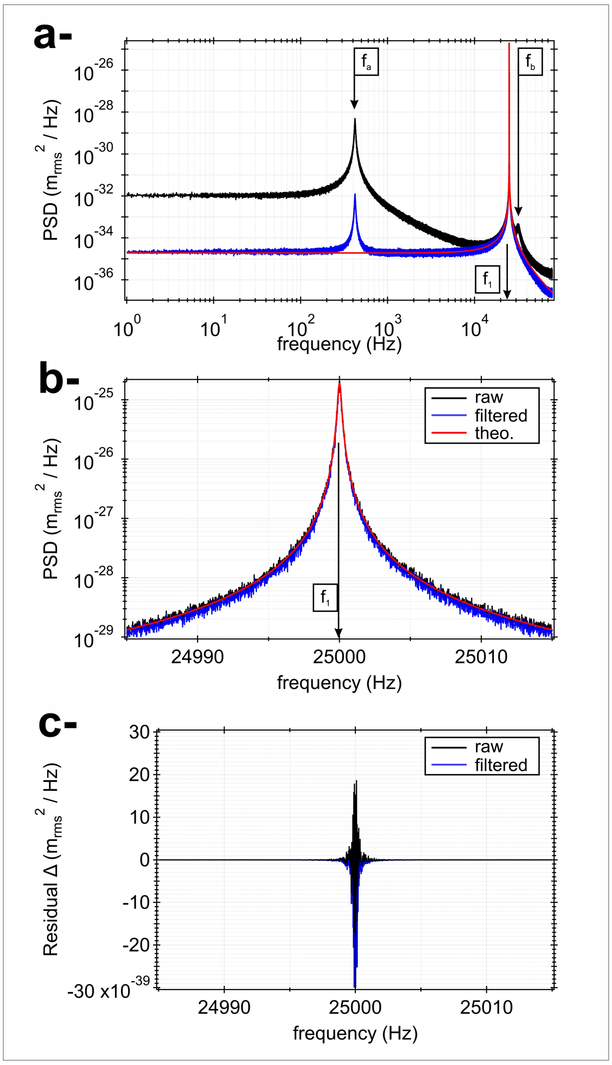

Figure S1: *a-Simulated tn-PSD spectrum at 9.8 K (spectral range:* $[0; B_s]$*) for the qPlus whose mechanical parameters are given in table 1 of the main text (black curve). The resonance peak due to the fundamental eigenmode (*f_1 = 25.000 *kHz) largely dominates the tn-PSD. Additional peaks (*f_a *and* f_b*) stemming from eigenmodes 2 and 3 are visible due to spectral aliasing (*cf. *text). An antialiasing filter attenuates those components (blue curve), yielding a noise floor then consistent with the theoretical tn-PSD (red curve). b-tn-PSD displayed in the spectral range* ±15 *Hz around* f_1 *(raw data: black curve, filtered data: blue curve, theoretical tn-PSD: red curve). c-Residuals calculated between the raw and the filtered tn-PSD and the theoretical tn-PSD (*cf. *text).*
